# Multi-objective optimization of ultrasound-assisted extraction for enhanced bioactive recovery from bee bread

**DOI:** 10.3389/fnut.2026.1970806

**Published:** 2026-09-24

**Authors:** Meryem Akhan, Enes Açikgözoğlu, Mehmet Ali Şimşek, Seydi Yıkmış, Remzi Gürfidan, Filiz Aksu, Sema Sandıkçı Altunatmaz, Melikenur Türkol Gündüz, Emad Karrar, Moneera O. Aljobair, Isam A. Mohamed Ahmed

**Affiliations:** 1 Department of Nutrition and Dietetics, Istanbul Esenyurt University, Istanbul, Türkiye; 2 Isparta Vocational School of Information Technologies, Cloud Computing Operator, Isparta University of Applied Sciences, Isparta, Türkiye; 3 Department of Software Engineering, Faculty of Engineering and Natural Sciences, Bandırma Onyedi Eylül University, Bandırma, Balikesir, Türkiye; 4 Department of Food Technology, Tekirdag Namik Kemal University, Tekirdag, Türkiye; 5 Department of Food Processing, Vocational School of Veterinary Medicine, Istanbul University-Cerrahpasa, Avcilar, Istanbul, Türkiye; 6 Nutrition and Dietetics, Faculty of Health Sciences, Tekirdag Namik Kemal University, Tekirdag, Türkiye; 7 Department of Plant Sciences, North Dakota State University, Fargo, ND, United States; 8 Department of Sports Health, College of Sports Sciences and Physical Activity, Princess Nourah bint Abdulrahman University, Riyadh, Saudi Arabia; 9 Department of Food Sciences and Nutrition, College of Food and Agricultural Sciences, King Saud University, Riyadh, Saudi Arabia

**Keywords:** bee bread, Gaussian process modeling, multi-objective Bayesian optimization, phenolic compounds, ultrasound-assisted extraction

## Abstract

This study aimed to optimize ultrasound-assisted extraction conditions for improving the recovery of phenolic compounds and antiradical activity from bee bread (perga). A three-factor Box–Behnken design comprising ultrasound amplitude (30–70%), extraction time (8–22 min), and ethanol concentration (20–80%, v/v) was combined with Gaussian Process surrogate modeling and a single-cycle GP-based multi-objective Bayesian optimization (GP-MOBO) candidate-selection strategy. Predictive uncertainty and Pareto-based analysis were used to identify a balanced extraction condition, which was subsequently validated experimentally and compared with an NUS and conventional extraction treatments. The selected ultrasound-assisted extraction condition (UAEopt; 56.5% amplitude, 19.0 min, and 56.8% ethanol) yielded a total phenolic content of 17.32 mg GAE/g, compared with 11.76 mg GAE/g in the matched NUS (*p* < 0.01). Antiradical activity also increased from 3.215 mg TEAC/g in NUS to 4.17 mg TEAC/g in UAEopt (*p* < 0.05). HPLC–DAD analysis showed that UAEopt produced the highest total concentration of the quantified phenolic compounds (44.19 ± 1.23 μg/g), with gallic acid as the predominant compound. LC–MS analysis further demonstrated that UAEopt yielded the highest total free amino acid concentration among the evaluated extraction conditions (23,409.32 ± 766.26 μg/g), with glutamic acid, aspartic acid, and proline as the major components. These findings demonstrate that Gaussian Process-guided multi-objective optimization can support the selection of effective ultrasound-assisted extraction conditions and improve the recovery of multiple bioactive fractions from bee bread.

## 1 Introduction

The increasing interest in natural and functional products today has made bee products an important component of scientific research and industrial applications due to their rich nutritional content and biological activities (1, 2). Bee pollen is formed when pollen collected by honey bees from flowers combines with nectar secreted by the bees and enzymatic components (3). This pollen, placed in the honeycomb cells of the hive, is transformed into a product called bee bread (perga) under the influence of honey, digestive enzymes, and organic acids released by the bees (4, 5). This transformation results from the fermentation of pollen by lactic acid bacteria and yeasts (6). Bee bread, a highly nutrient-dense bee product, contains significant amounts of carbohydrates, proteins, and lipids, as well as vitamins, minerals, phenolic compounds, and essential amino acids. This composition gives bee bread its versatile biochemical and functional properties (7, 8).

Bee bread contains numerous bioactive compounds, including phenolic compounds associated with antioxidant properties (9). Phenolic compounds, particularly polyphenols, are antioxidants that play a fundamental role in protecting biomolecules from oxidative damage by scavenging reactive oxygen and nitrogen species. This effect occurs primarily through mechanisms based on hydrogen atom and electron transfer, resulting in the formation of more stable and lower-reactive products. These compounds, which cannot be synthesized by humans or animals, are important bioactive components that must be obtained through diet (10, 11). The significant antioxidant potential of bee bread is largely attributed to its polyphenolic compounds, making it a functional natural antioxidant source (12–14).

Ultrasound extraction provides the necessary theoretical background and some details regarding techniques and combinations thereof, mechanisms (fragmentation, erosion, capillarity, tissue disruption, and sonoporation), applications from the laboratory to industry, safety, and environmental impacts. Owing to these features, UAE has become a prominent research topic across multiple domains of plant-based chemistry. Accumulating evidence indicates that UAE represents a green and economically viable alternative to conventional extraction methods for food and natural products, offering advantages in terms of process efficiency, environmental sustainability, and operational safety (15–18). Recent studies have demonstrated the effectiveness of ultrasound-based techniques in recovering and preserving plant bioactives. Tahir et al. (19) highlighted that UAE enhances polyphenol recovery from fruit peels through cell-wall disruption, while Muntaha et al. (20) experimentally showed that UAE outperformed microwave, Soxhlet, and maceration methods in extracting bioactive compounds from orange pomace. Similarly, Altarjami (21) reported that hybrid ultrasound–microwave extraction improved polyphenol recovery from pomegranate peel and produced extracts with strong antioxidant and anticancer activities. Beyond extraction, Dülger Altıner et al. (22) demonstrated that machine learning-optimized ultrasound–ohmic processing enhanced the phenolic profile and antidiabetic potential of hawthorn vinegar.

Machine learning (ML) approaches have recently emerged as powerful tools in reaction computing (23). System design is becoming increasingly complex under constantly changing operating conditions. The need to simultaneously meet numerous performance criteria, coupled with increasing design freedom, is driving a demand for multi-objective optimization (MOO) methods that reduce reliance on expertise and utilize algorithm-based approaches and virtual prototyping. Gaussian process regression is frequently used as a surrogate model in multi-objective Bayesian optimization frameworks, providing an efficient Pareto-front approach to complex design problems such as power system optimization (24).

In this context, the present study aimed to model the nonlinear and interactive relationships between ultrasound-assisted extraction parameters and key antioxidant-related responses of bee bread, namely Total Phenolic Content (TPC) and DPPH free radical scavenging activity. To this end, Gaussian Process (GP) regression was employed as a probabilistic surrogate modeling approach due to its strong capability to capture complex nonlinear response behavior while simultaneously providing predictive uncertainty estimates. Building on these surrogate models, a single-cycle GP-MOBO candidate-selection framework was implemented to balance exploration and exploitation in the joint maximization of TPC and DPPH. The approach was selected because probabilistic surrogate modeling allows predicted responses and predictive uncertainty to be considered jointly when identifying candidate operating conditions from a limited experimental dataset. To critically assess the effectiveness of the proposed strategy, its performance was systematically compared with random sampling and commonly used surrogate-based metaheuristic algorithms, including genetic algorithm (GA), particle swarm optimization (PSO), and whale optimization–based approaches. Such comparative evaluation is particularly important in multi-objective systems, as different optimization strategies may exhibit varying strengths in approximating Pareto-optimal solutions for individual or combined responses. By integrating surrogate modeling, uncertainty quantification, and comparative optimization analysis, this study provides a comprehensive framework for guiding experimental extraction design toward high-quality antioxidant performance.

## 2 Materials and methods

Bee bread was collected from 12 independent beekeepers in Tekirdağ Province (Türkiye). Equal masses from each producer were pooled to obtain a composite sample to improve representativeness. Ethanol (EMSURE^®^, Reag. Ph. Eur.; 95.1–96.9% v/v; Merck KGaA, Darmstadt, Germany; product no. 1.59010/159010) and ultrapure/distilled water were used to prepare aqueous ethanol (hydroalcoholic) extraction solvents, which were obtained by volumetric dilution of the 96% (v/v) stock ethanol with water to the desired ethanol ratios (% v/v). Total phenolic content (TPC) was determined using the Folin–Ciocalteu reagent with gallic acid as the calibration standard. Antiradical activity was evaluated by the DPPH free radical scavenging assay using Trolox as the calibration standard. All other chemicals were of analytical grade. Unless otherwise stated, consumables (centrifuge tubes, filters, cuvettes) were single-use.

### 2.1 Sample preparation

The pooled bee bread was homogenized by grinding/milling to a uniform particle size, mixed thoroughly, placed in airtight containers protected from light, and stored at −20 °C until extraction. Prior to each extraction, samples were equilibrated to room temperature and mixed to ensure homogeneity. No drying step was applied prior to extraction; all extractions were performed using the non-dried material (as received). To document the water content of the material used for extraction, moisture content of the homogenized bee bread was determined in triplicate using a gravimetric oven-drying approach according to AOAC: approximately 2.0 g of sample was dried at 105 °C to constant mass, and moisture (%) was calculated from mass loss (25). All results are reported on an as-received (wet-weight) basis.

### 2.2 Extraction procedure and experimental groups

Extractions were carried out at a constant solid-to-liquid ratio of 1:20 (w/v) (1.00 g bee bread + 20.0 mL solvent). The solvent composition used for the comparative experiments followed the representative Pareto-optimal operating condition selected for reporting: 56.8% (v/v) ethanol in water. Four extraction arms were considered: (i) UAEopt, ultrasound-assisted extraction performed at the representative operating condition selected from the GP-MOBO candidate set; (ii) NUS, no-ultrasound control using the same solvent composition and contact time as UAEopt; (iii) CE60, conventional extraction by orbital shaking for 60 min; and (iv) CE120, conventional extraction by orbital shaking for 120 min. In CE arms, the same solvent composition and solid-to-liquid ratio were maintained.

UAEopt was performed using a probe-type ultrasonic processor (Hielscher Ultrasonics UP200St; 26 kHz; 200 W). The ultrasonic processor was equipped with a 10 mm-diameter probe and operated in continuous mode. Each 20.0 mL extraction mixture was processed in a cylindrical glass jar with a nominal capacity of 100 mL. The probe was positioned centrally, with its tip immersed to approximately the mid-depth of the liquid, without contacting the walls or bottom of the jar. The reported 200 W represents the nominal maximum output power of the device. The actual acoustic power delivered to the sample was not determined calorimetrically; therefore, acoustic power density (W/mL) and specific energy input (J/mL) could not be calculated. Sonication was conducted at 56.5% amplitude for 19.0 min in 56.8% (v/v) ethanol. To minimize thermal artifacts, the extraction vessel was externally cooled using an ice-water bath to limit the temperature increase during sonication. However, we did not record either the initial or final temperature of the extraction mixture, and we did not continuously monitor the ice-bath temperature. Consequently, the temperature increase during sonication and mean ± SD temperature values cannot be reported retrospectively. This is a methodological limitation, as the contribution of ultrasound-induced heating to the observed extraction responses cannot be fully distinguished from the non-thermal effects of sonication. For NUS, the mixture was processed for 19.0 min under identical solvent conditions without applying ultrasound, using constant agitation to maintain mass transfer. Conventional extractions (CE60/CE120) were carried out at 25 ± 1 °C and 150 rpm. After extraction, suspensions were clarified by centrifugation at 4,000 rpm for 10 min. Supernatants were collected and filtered through Whatman No. 41 filter paper prior to chemical analyses. A three-factor response dataset (Table 1) was generated to model the effects of ultrasound amplitude (%), extraction time (min), and ethanol concentration (% v/v) on TPC and DPPH responses. Factor levels were coded to the range −1 to + 1 for surrogate modeling, and measured responses were used as training targets for the optimization framework. Each row in Table 1 represents a separately prepared extraction performed under the specified experimental conditions. The three center-point runs were conducted as independent extractions to estimate experimental variability and pure error. TPC and DPPH analyses were performed in triplicate for each extract as analytical replicates, and the mean of these three measurements was used as the response value for modeling. Analytical replicates were not treated as independent experimental runs.

**TABLE 1:** Experimental design matrix (coded and real factor levels) and measured responses (TPC and DPPH).

Exp. No	X_1_ (Code)	X_2_ (Code)	X_3_ (Code)	Amplitude (%)	Time (min)	Ethanol (%)	TPC (mg GAE/g)	DPPH (mg TEAC/g)
1	1	0	−1	70	15	20	13.86	3.12
2	−1	0	1	30	15	80	14.96	3.35
3	0	−1	−1	50	8	20	14.05	2.94
4	1	−1	0	70	8	50	14.67	3.54
5	0	0	0	50	15	50	16.43	3.76
6	0	1	1	50	22	80	15.43	3.86
7	−1	−1	0	30	8	50	14.91	3.27
8	0	1	−1	50	22	20	15.94	3.18
9	0	−1	1	50	8	80	16.12	3.45
10	1	1	0	70	22	50	15.84	3.95
11	1	0	1	70	15	80	16.55	3.70
12	−1	1	0	30	22	50	15.38	3.69
13	0	0	0	50	15	50	16.43	3.76
14	−1	0	−1	30	15	20	15.72	2.77
15	0	0	0	50	15	50	16.43	3.76

X_1_, Amplitude (%); X_2_, Time (min); X_3_, Ethanol Concentration (%); TPC, Total Phenolic Content (mg GAE/g); GAE, Gallic Acid Equivalent; DPPH, 2,2-Diphenyl-1-Picrylhydrazyl Radical Scavenging Capacity (mg TEAC/g); TEAC, Trolox Equivalent Antiradical activity. Each row represents an independently prepared extraction. TPC and DPPH were measured in triplicate as analytical replicates for each extract, and the mean value was used for statistical modelling.

In the design, three independent variables (Amplitude, Duration, Ethanol ratio) are given both at coded levels (X_1_, X_2_, X_3_) and in real units, ensuring that the variables are brought to a comparable scale during the modeling phase. The independent variables were defined as X_1_ (Amplitude), X_2_ (Duration), X_3_ (Ethanol ratio); in the experimental design, each variable was considered at three levels, using the levels −1, 0, + 1 in the coded space (e.g., Amplitude: 30–50–70; Duration: 8–15–22; Ethanol: 20–50–80). For each experimental condition, the response variables Total Phenolic Content (TPC, mg GAE/g) and the antiradical activity indicator DPPH (mg TEAC/g) were measured and reported in Table 1. The repeated testing of the center point (0,0,0) in the design enabled the estimation of experimental repeatability and measurement noise (pure error), making the GP model’s uncertainty estimates more reliable. The repeated application of the center point is a critical control mechanism for estimating experimental noise/pure error and evaluating the reliability of the model. This table enhances the traceability of the method and results as it directly defines the observational data set on which the surrogate model (GP) was trained.

### 2.3 Total phenolic content (TPC)

The Folin–Ciocalteu assay was employed to quantify the TPC (26). For extract preparation, 2 mL of sample was mixed with 8 mL of 80% (v/v) methanol (1:5, v/v), vortexed, and centrifuged at 4,000 rpm for 20 min to remove insoluble material and suspended particles. The clarified methanolic extract was used for analysis. The extract was further diluted with 80% (v/v) methanol prior to the Folin–Ciocalteu assay to obtain a homogeneous analytical solution suitable for spectrophotometric measurement and to ensure that the absorbance values remained within the linear range of the gallic acid calibration curve. This additional dilution was taken into account in the calculation of TPC using the corresponding dilution factor. Briefly, 50 μL of the clarified extract was mixed with 100 μL of Folin–Ciocalteu reagent (Sigma-Aldrich, F9252), and the volume was made up to 1,450 μL with distilled water. After 10 min at room temperature, 50 μL of 7.5% (w/v) Na_2_CO_3_ was added (final volume 1,500 μL). The mixture was incubated in the dark for 2 h, and absorbance was measured at 765 nm. TPC was expressed as milligrams of gallic acid equivalents per gram of bee bread (mg GAE/g).

### 2.4 DPPH free radical scavenging activity

The antioxidant activity was determined using the 2,2-diphenyl-1-picrylhydrazyl (DPPH●) radical scavenging assay (27). Briefly, 1 mL of bee bread sample was mixed with 1 mL of DPPH● solution (0.2 mM in methanol). The reaction mixture was incubated for 30 min at room temperature (25 ± 1 °C) in the dark. Absorbance was measured at 517 nm using a UV–Vis spectrophotometer (SP-UV/VIS-300SRB, Spectrum Instruments, Melbourne, Australia). Experimental design and response dataset.

DPPH● radical scavenging activity was calculated using Equation 1:


DPPH•radicalscavengingactivity(%)=(A0−A1)/A0x 100 (1)

*A_0_* represented the absorbance of the control sample, while *A_1_* represented the absorbance measured for the bee bread samples.

### 2.5 Analysis of phenolic compounds (HPLC)

Chromatographic separation was carried out on an ACE Genix C18 column (250 × 4.6 mm, 5 μm; Agilent), following the procedure reported by Portu et al. (28). Phenolics were characterized using an Agilent 1260 HPLC–DAD system operated under gradient conditions at 30 °C with a flow rate of 0.80 mL/min. The mobile phase comprised solvent A (water supplemented with 0.1% phosphoric acid) and solvent B, applied according to a predefined gradient program. Samples were injected at 10 μL, and chromatograms were recorded at 280, 320, and 360 nm. Quantitative results were reported as μg/mL. The complete list of phenolic standards, detailed chromatographic parameters (including the full gradient program), and representative chromatograms for all extraction conditions (A = NUS, B = CE60, C = CE120, D = UAEopt).

### 2.6 Determination of free amino acids (LC-MS)

Amino acid composition was determined using a method adapted with minor modifications from the procedure described by Bilgin et al. (29). A gradient elution scheme involving mobile phases A and B was applied to obtain chromatographic separation at a flow rate of 0.7 mL/min, yielding a total run duration of 7.5 min. Tandem mass spectrometric analyses were performed on an Agilent 6460 triple quadrupole LC–MS instrument (Agilent Technologies, Waldbronn, Germany) fitted with an electrospray ionization (ESI) interface. The mass spectrometer was operated with a gas temperature of 150 °C, a gas flow rate of 10 L/min, a nebulizer pressure of 40 psi, and a capillary voltage of + 2,000 V. All analyses were carried out in triplicate, and the results were reported as mg/100 mL.

### 2.7 Gaussian process modeling and single-cycle multi-objective Bayesian optimization (GP-MOBO)

In this study, a surrogate-based optimization framework was adopted to evaluate the effects of three process variables (Amplitude, Duration, and Ethanol ratio) on total phenolic content (TPC) and antiradical activity indicator (DPPH) during the bee bread extraction process, and to determine the conditions that simultaneously maximize both responses. The ranges of the independent variables were selected based on preliminary trials to ensure stable cavitation and practical operation without excessive foaming/overheating, and were further aligned with commonly reported UAE parameter windows for hydroalcoholic extraction of phenolics from complex food matrices. Accordingly, the experimental design was implemented using a Box–Behnken design with factor levels set at 30–50–70% for Amplitude, 8–15–22 min for Time, and 20–50–80% (v/v) for Ethanol. The primary objective of the optimisation was to maximize the two measured responses (TPC and DPPH) simultaneously. To this end, a separate Gaussian Process Regression (GPR) model was established for each response (Kriging), yielding a data-driven predictor capable of representing uncertainty quantitatively, without constraining the factor-response relationships with a parametric polynomial.

Gaussian Process (GP) regression was implemented following the probabilistic framework described by Rasmussen and Williams, where the response function is modeled as a Gaussian process defined by a mean function and a covariance kernel. The Matérn kernel (*v* = 2.5) was selected due to its flexibility in modeling moderately smooth nonlinear functions and its frequent use in surrogate modeling and Bayesian optimization applications (30, 31).

The multi-objective Bayesian optimization strategy was inspired by the Expected Improvement (EI) acquisition function originally introduced by Jones et al. and extended to multi-objective settings using scalarization approaches (32). A ParEGO-style scalarization strategy was adopted, using randomly sampled objective-weight vectors, augmented Chebyshev scalarization, and Expected Improvement (33).

All computational analyses were conducted in Python (version 3.14.0) using the scikit-learn library for Gaussian Process regression, including Matérn kernels and White Kernel noise modeling. Hyperparameters were optimized by maximizing the marginal log-likelihood. The Bayesian optimization workflow, including scalarization and Expected Improvement computation, was implemented using custom Python routines to ensure full control over the surrogate–acquisition interaction and evaluation budget.

Gaussian Process regression was implemented in Python 3.14.0 using scikit-learn version 1.9.0. Separate GP models were fitted for TPC and DPPH in the coded factor space (−1 to + 1). The covariance function was defined as the product of a ConstantKernel and an anisotropic Matérn kernel, together with an additive WhiteKernel noise term:


$$k\,\left(x,x^{\prime }\right)=\sigma_{f}^{2}\,k_{\,{\mathrm{Matern}}^{\prime }5/2}\,\left(x,x^{\prime }\right)+\sigma_{n}^{2}\,\delta_{x,x^{\prime }},$$

where


$$k_{\,{\mathrm{Matern}}^{\text{’}}5/2}=$$

$$\left(1+\sqrt{5}\,r+\frac{5\,r^{2}}{3}\right)\,\exp\,\left(-\sqrt{5}\,r\right),r=\sqrt{\sum_{j=1}^{3}{\left(\frac{x_{j}-x_{j}^{\prime }}{\ell_{j}}\right)}^{2}},$$

and ℓ_*j*_ denotes an independent length-scale parameter for each of the three extraction factors. The ConstantKernel was initialized at 1.0 with bounds of 10^−3^to 10^3^; each Matérn length scale was initialized at 1.0 with bounds of 10^−2^to 10^2^; and the WhiteKernel noise level was initialized at 10^−5^with bounds of 10^−8^to 10^−1^. No additional diagonal nugget was introduced (α = 0), and target normalization was enabled (normalize_y = True). Kernel hyperparameters were estimated by maximizing the log-marginal likelihood using the default L-BFGS-B optimizer implemented in GaussianProcessRegressor. To reduce sensitivity to local optima, 30 optimizer restarts were performed (n_restarts_optimizer = 30) in addition to the initial optimization run. Reproducibility was controlled using fixed pseudo-random number generation with a global seed of 7; deterministic derived random streams were used for final GP fitting, cross-validation, candidate generation, and optimization-method comparison.

When TPC and DPPH are to be maximized simultaneously, instead of a single “absolute optimum,” a set of Pareto-optimal solutions emerges that can vary depending on the decision-maker’s priorities. Therefore, in the first stage, a Pareto dominance analysis was performed using only the observed experimental points. A point is classified as non-dominated (Pareto-optimal) if it is not simultaneously dominated by another point in terms of both TPC and DPPH (i.e., it is at least as good as the other point in one aspect and at least equal in the other). This analysis reveals the best equilibrium points already present in the data and provides an assessment appropriate to the nature of the multi-objective problem.

In the second stage, GP models were used to generate predictions for points in the experimental space that had not yet been measured (in the coded space −1 + 1), and the “predicted Pareto front” was calculated. This allowed the exploration of multi-objective improvement potential across the continuous space permitted by the model, without being constrained to just 15 experimental points. The ParEGO-style scalarization strategy was used to convert the multi-objective problem into a sequence of scalar sub-problems defined by different objective-weight vectors (w_TPC, w_DPPH), with each weight vector targeting a different region of the Pareto front. By using the augmented Chebyshev function in the scaling (adding a small total term with ρ), the solutions are prevented from becoming overly concentrated on a single objective; thus, a more balanced sampling across the Pareto front is targeted.

The distinctive aspect of the optimization in this study is that it not only maximizes the predicted mean values but also proposes new experimental conditions using an acquisition function that accounts for model uncertainty. For each candidate point X, the GP model approximates the posterior distribution of TPC and DPPH as a Normal distribution (mean μ and standard deviation σ). After the multi-objective problem is scaled, the concept of “improvement” is defined as the expected gain relative to the best value currently available in the scaled objective function. To this end, Expected Improvement (EI) was calculated using Monte Carlo sampling; points with high EI values represented regions with both high output potential and uncertainty, making them worth exploring. Consequently, the proposed candidate points were selected not only according to favorable posterior mean predictions but also by accounting for predictive uncertainty. This allowed the acquisition procedure to prioritize candidate conditions with potentially favorable response–uncertainty trade-offs. This allowed the acquisition procedure to prioritize candidate conditions with potentially favorable response–uncertainty trade-offs. This approach is useful for prioritizing candidate conditions when the available experimental dataset is limited.

Gaussian process (GP) surrogate models were fitted separately for TPC and DPPH to capture nonlinear relationships between process variables and responses. Model predictive accuracy and uncertainty calibration were evaluated by leave-one-out cross-validation (LOOCV) using MAE/RMSE for point prediction and coverage-based metrics for uncertainty (as detailed in the Results section).

It should be emphasized that the present study employed a single GP-guided acquisition cycle rather than a fully sequential closed-loop Bayesian optimization campaign. The initial 15 Box–Behnken observations were used to fit the GP surrogate models, after which the acquisition procedure generated eight candidate conditions. One representative candidate (S4) was selected and subsequently validated experimentally. The experimentally measured S4 response was not fed back into the GP models to initiate another surrogate-update/acquisition cycle. Accordingly, GP-MOBO in the present study refers to a single-cycle surrogate-assisted multi-objective candidate-selection framework rather than fully iterative experimental Bayesian optimization.

Because the optimization is inherently multi-objective (simultaneous maximization of TPC and DPPH), the solution is a set of Pareto-optimal operating conditions rather than a single global optimum. For reporting and for the comparative extraction experiment, one representative condition was selected from the Pareto front where both objectives remained high without disproportionately sacrificing either response. According to GP-MOBO recommendations, the representative operating condition (S4) corresponded to approximately 56.5% amplitude, 19.0 min extraction time, and 56.8% (v/v) ethanol. Under these conditions, the GP posterior mean estimates were 16.18 mg GAE/g for TPC and 3.96 mg TEAC/g for DPPH.

A fully sequential closed-loop Bayesian optimization framework could further improve this process by experimentally evaluating the selected candidate, updating the GP surrogate with the newly measured response, and repeating the acquisition–validation cycle until a predefined convergence or stopping criterion is reached. Such an iterative strategy may progressively refine the Pareto front and reduce predictive uncertainty in promising regions of the design space.

For the surrogate-level comparison, the hyperparameters of all metaheuristic algorithms were fixed a priori and kept unchanged across independent runs. The genetic algorithm (GA) employed a population size of 40, a crossover probability of 0.90, and a mutation probability of 0.20. Gaussian perturbation was used for mutation with a standard deviation of 0.12 in the coded factor space. Particle swarm optimization (PSO) used a swarm size of 40, an inertia weight of *w* = 0.72, a cognitive acceleration coefficient of *c*_1_ = 1.49, and a social acceleration coefficient of *c*_2_ = 1.49. Whale optimization algorithm (WOA) employed 40 search agents. Its control parameter decreased linearly according to *a* = 2−2*t*/*T*, where *t* and *T* denote the current and maximum update iterations, respectively. The WOA coefficient vectors were calculated as *A* = 2*ar*_1_−*a* and *C* = 2*r*_2_, where *r_1_* and *r_2_* were independently sampled from *U*(0,1). For the spiral-search component, *p*∼*U*(0,1) and *l*∼*U*(−,11). Candidate solutions for all algorithms were constrained to the coded three-dimensional design domain [–, + 1]^3^. GA, PSO, and WOA each used an initial population/swarm of 40 followed by two update iterations, resulting in a total computational budget of 120 surrogate-function evaluations per run. Each algorithm was independently repeated 20 times using deterministic pseudo-random streams derived from the global seed of 7.

### 2.8 Gaussian process validation and uncertainty calibration (LOOCV)

To evaluate the out-of-sample predictive performance and uncertainty calibration of the Gaussian Process (GP) surrogate models, leave-one-out cross-validation (LOOCV) was performed separately for TPC and DPPH. The three experimental factors were represented in the coded Box–Behnken design space ranging from −1 to + 1, and no additional fold-specific standardization of the predictor variables was applied. For each LOOCV fold, one experimental observation was excluded from the dataset, and a new GP model was fitted using the remaining *n-1*observations. The same covariance structure, initial hyperparameter values, parameter bounds, and optimization settings described in section 2.7 were used in every fold, while the kernel hyperparameters were independently re-optimized by maximizing the log-marginal likelihood. Target normalization was enabled during model fitting (normalize_y = True). This procedure ensured that the prediction for each held-out observation was generated by a GP model that had not been trained using that observation. The implementation fitted a completely new GP model at every LOOCV fold.

For each held-out observation *i*, the fitted GP returned a posterior predictive mean μ_*i*_and posterior predictive standard deviation σ_*i*_. Point-prediction performance was assessed using mean absolute error (MAE), root mean squared error (RMSE), and the coefficient of determination (*R*^2^):


$$M\,A\,E=\frac{1}{n}\,\overset{n}{\underset{i=1}{\sum }}|y_{i}-{\hat{y}}_{i}|$$

$$R\,M\,S\,E=\sqrt{\frac{1}{n}\,\overset{n}{\underset{i=1}{\sum }}{\left(y_{i}-{\hat{y}}_{i}\right)}^{2}}$$

$$R^{2}=1-\frac{\sum_{i=1}^{n}{\left(y_{i}-{\hat{y}}_{i}\right)}^{2}}{\sum_{i=1}^{n}{\left(y_{i}-\bar{y}\right)}^{2}}$$

where *y_i_* is the experimentally observed response, ${\hat{y}}_{i}$ is the corresponding LOOCV prediction, and $\bar{y}$ is the mean of the observed responses. The cross-validated *R*^2^ values were retained without truncation, sign correction, or absolute-value transformation; therefore, negative values were allowed when the LOOCV predictions performed worse than the observed-response mean used as a reference benchmark. This calculation was additionally verified against the explicit 1−*SSE*/*SST* formulation in the computational workflow.

Predictive uncertainty was evaluated using prediction interval coverage probability (PICP) and mean prediction interval width (MPIW) at nominal coverage levels of 90% and 95%. Approximate two-sided GP predictive intervals were calculated from the posterior predictive mean and standard deviation as:


$$P\,I_{1-\alpha }\,\left(x_{i}\right)=\left[\mu_{i},-z_{1-\alpha /2}\,\sigma_{i}\,\mu_{i}+z_{1-\alpha /2}\,\sigma_{i}\right]$$

where *z*_*1–α/2*_represents the corresponding standard-normal critical value. Values of *z* = 1.645 and *z* = 1.960 were used for the 90 and 95% prediction intervals, respectively. The implementation explicitly used the two-sided critical value *z*_*1–α /2*_.

Prediction interval coverage probability was calculated as:


$$P\,I\,C\,P=\frac{1}{n}\,\overset{n}{\underset{i=1}{\sum }}I\,\left(L_{i}\leq y_{i}\leq U_{i}\right)$$

where *L_i_* and *U_i_* denote the lower and upper prediction limits, respectively, and *I*(⋅) is an indicator function equal to 1 when the observed response falls inside the prediction interval and 0 otherwise. The mean prediction interval width was calculated as:


$$M\,P\,I\,W=\frac{1}{n}\,\overset{n}{\underset{i=1}{\sum }}\left(U_{i}-L_{i}\right)$$

PICP therefore quantifies how frequently the observed responses are contained within the nominal GP predictive intervals, whereas MPIW characterizes the average uncertainty-band width. Both metrics were calculated independently for the 90% and 95% nominal coverage levels.

To further assess uncertainty calibration, standardized residuals were calculated for each held-out observation as:


$$r_{i}^{*}=\frac{y_{i}-\mu_{i}}{\sigma_{i}}$$

and their distribution was examined across all LOOCV predictions. A standardized residual distribution approximately centered around zero with a dispersion close to one indicates better agreement between the magnitude of the GP predictive uncertainty and the actual prediction errors. Conversely, a dispersion substantially greater than one suggests that predictive uncertainty may be underestimated, whereas a substantially lower dispersion may indicate overly conservative uncertainty estimates. The resulting MAE, RMSE, *R*^2^, PICP, MPIW, and standardized-residual statistics were used jointly to evaluate both predictive accuracy and uncertainty calibration of the TPC and DPPH surrogate models.

### 2.9 Statistical analysis

All measurements were performed at least in triplicate unless otherwise stated. Results are presented as mean ± standard deviation. Where applicable, differences among extraction arms were assessed by one-way analysis of variance (ANOVA) followed by a suitable post hoc test, with *p* < 0.05 considered statistically significant.

## 3 Results and discussion

### 3.1 Comparisons of optimization methods

In this study, the Surrogate-Based Multi-Objective Bayesian Optimization (GP-MOBO) approach was employed on a GP-based surrogate for a two-objective (TPC and DPPH) maximization problem; additionally, to demonstrate the robustness of the decision, three meta-heuristic methods (GA, PSO, and WOA) were compared with random sampling under the same evaluation budget. The comparison was made using metrics that measure the overall quality of the Pareto front rather than a single “best point” and are standard in multi-objective optimization: the number of non-dominated solutions (Pareto Points), the hypervolume (HV) representing the area covered by the Pareto front, and the highest values achieved for each objective (Max_TPC- Maximum total phenolic content, Max_DPPH- maximum free radical scavenging activity). When these metrics are evaluated together, it is possible to quantitatively report not only whether the methods reached the maximum, but also the diversity of the solutions and the overall quality of the front. Table 2 summarizes the results of this comparison.

**TABLE 2:** Comparison of different methods (GP-MOBO, random sampling, and meta-heuristic approaches) in terms of Pareto frontier quality (HV) in two-objective optimization (maximizing TPC and DPPH); HV values are reported in a normalized objective space.

Metric	GP-MOBO	Random	WOA	PSO	GA
HV_mean	0.970004	0.920329	0.932346	0.939238	0.912000
HV_std	0.002507	0.022435	0.021055	0.016879	0.019620
ParetoPoints_mean	40.400000	8.050000	22.000000	13.900000	18.850000
ParetoPoints_std	3.589458	1.538112	4.790341	2.531902	5.274317
Max_TPC_mean	16.548854	16.456612	16.467842	16.483566	16.438517
Max_TPC_std	0.005850	0.056128	0.026762	0.046669	0.043477
Max_DPPH_mean	4.061492	4.025212	4.034621	4.040659	4.012653
Max_DPPH_std	0.001712	0.020197	0.029989	0.021928	0.024940

GP-MOBO, Gaussian Process-based Multi-Objective Bayesian Optimization; GP, Gaussian Process; GA, Genetic Algorithm; PSO, Particle Swarm Optimization; WOA, Whale Optimization Algorithm; Random, random sampling; HV, hypervolume; ParetoPoints, number of non-dominated solutions; Max_TPC, highest total phenolic content obtained; Max_DPPH, highest DPPH response obtained; TPC, total phenolic content (mg GAE/g); GAE, gallic acid equivalent; DPPH, DPPH free radical scavenging capacity (mg TEAC/g); TEAC, Trolox equivalent antiradical activity; mean, average; std, standard deviation.

We used the dominated hypervolume (HV) as the primary indicator of multi-objective optimization performance, computed over the non-dominated set obtained at each evaluation budget. Since both objectives (TPC and DPPH) are defined as maximization problems and expressed in different physical units, they were first mapped to a normalized space prior to HV computation in order to ensure scale consistency and comparability.

For each objective *f*_*i*_(*x*), the normalized value was computed using min–max scaling:


$${\tilde{f}}_{i}\,\left(x\right)=\frac{f_{i}\,\left(x\right)-f_{i}^{\min}}{f_{j}^{\max}-f_{j}^{\min}},f\,o\,r\,j=1,2.$$

For hypervolume-based method comparison, a common normalization frame was fixed before comparing the search strategies. A dense reference set was uniformly sampled over the coded design space and evaluated using the fitted GP surrogate means. The resulting objective-wise minimum and maximum values were used as fixed normalization bounds for all optimization methods.


$$\tilde{f_{j}}\,\left(x\right)=\frac{f_{j}\,\left(x\right)-f_{j}\,m\,i\,n}{f_{j}\,\max-f_{j}\,m\,i\,n}$$

$$g_{j}\,\left(x\right)=1-\tilde{f_{j}}\,\left(x\right)$$

r=(1,1)

The same fixed normalization bounds and the same reference point were used for GP-MOBO, GA, PSO, WOA, and Random sampling. Because the transformed objectives were evaluated within the normalized unit square, the resulting two-dimensional hypervolume was bounded by 0 < = HV < = 1.

After normalization, both objectives lie within the interval 0|1], thereby eliminating scale effects and enabling direct comparison.

Because the hypervolume indicator is conventionally defined for minimization problems, the normalized maximization objectives were transformed as:


$$g_{i}\,\left(x\right)=1-{\tilde{f}}_{i}\,\left(x\right),f\,o\,r\,i=1,2.$$

so that smaller values correspond to better performance. In the transformed minimization space, the reference point was defined as r=(1,1) w, ch represents the worst attainable point after normalization. The dominated hypervolume was then computed as the Lebesgue measure of the region bounded by the non-dominated solution set *P*and the reference point:


$$H\,V\,\left(P\right)=\lambda \,\left({⋃}_{x\in P}\left[g_{1}\,\left(x\right),r_{1}\right]\times \left[g_{2}\,\left(x\right),r_{2}\right]\right)$$

where λ(⋅) denotes the two-dimensional Lebesgue measure. For methodological consistency, the same normalization constants $f_{i}^{\min}$, $f_{i}^{\max}$, and the same reference point r r were used across all optimization methods (GP-MOBO, GA, PSO, WOA, and random sampling), ensuring that differences in HV values reflect genuine differences in Pareto-front quality rather than scaling artifacts.

For the random sampling baseline, candidate solutions were generated by independent and identically distributed (i.i.d.) uniform sampling within the continuous design space defined by the coded factor bounds (x_1_, x_2_, x_3_ ∈ [−1, + 1]). At each evaluation budget, points were drawn from a three-dimensional uniform distribution over this domain without surrogate guidance or adaptive refinement. The same total number of function evaluations (NFE) and stopping criteria were used for all optimization methods to ensure a fair and controlled comparison.

To ensure a fair surrogate-level comparison, all search methods were constrained to an identical computational budget of 120 surrogate-function evaluations (NFE) per independent run. GA used a population size of 40; after the initial population evaluation, two evolutionary update generations of up to 40 candidates each were performed, yielding a total of 120 surrogate evaluations. PSO similarly employed 40 particles and two swarm-update iterations following the initial swarm evaluation, resulting in 120 evaluations. WOA used 40 search agents and two population-update iterations after initialization, likewise, resulting in 120 evaluations. Random sampling generated exactly 120 independently and uniformly sampled candidate points within the coded design space. The GP-guided search was initialized with 12 random evaluations and subsequently performed 108 sequential acquisition-guided evaluations, resulting in the same total budget of 120 NFE. Each method was independently repeated 20 times using deterministic but distinct pseudo-random number generator streams derived from a global seed of 7. The implementation explicitly verified that each method returned exactly 120 evaluated surrogate points in every repeat; otherwise, execution was terminated with an error. Therefore, all methods were compared under an identical surrogate-evaluation budget. Computational settings used for the surrogate-level optimization-method comparison are shown in Table 3.

**TABLE 3:** Computational settings used for the surrogate-level optimization-method comparison.

Method	Population/swarm	Update iterations	Algorithm-specific hyperparameters	Total NFE/run	Runs
GP-guided	–	108 acquisitions	12 initial random evaluations	120	20
Random	–	–	Uniform sampling in −,1+1]^3^	120	20
GA	40	2	*p*_*c*_ = 0.90; *p*_*m*_ = 0.20; Gaussian mutation SD = 0.12	120	20
PSO	40	2	*w* = 0.72; *c*_1_ = 1.49; *c*_2_ = 1.49	120	20
WOA	40	2	*a* = 2−2*t*/*T*; *A* = 2*ar*_1_−*a*; *C* = 2*r*_2_; *p*∼*U*(0,1); *l*∼*U*(−,11)	120	20

NFE, number of fitted-surrogate function evaluations; GA, genetic algorithm; PSO, particle swarm optimization; WOA, whale optimization algorithm. For WOA, *r*_1_,*r*_2_∼*U*(0,1). All candidate solutions were constrained to the coded domain [−,1+1]^3^. A global random seed of 7 was used to generate deterministic random streams for independent runs. NFE represents computational surrogate evaluations and does not correspond to additional laboratory experiments.

The algorithm-specific control parameters were also fixed across all independent runs. For GA, the population size was 40, the crossover probability was 0.90, the mutation probability was 0.20, and Gaussian mutation noise was applied with a standard deviation of 0.12 in the coded factor space. For PSO, a swarm size of 40 particles was used with an inertia weight of 0.72, a cognitive coefficient of *c*_1_ = 1.49, and a social coefficient of *c*_2_ = 1.49. For WOA, 40 search agents were used, and the control parameter was updated according to *a* = 2−2*t*/*T*. The coefficient vectors were calculated as *A* = 2*ar*_1_−*a*and *C* = 2*r*_2_, with *r_1_*and *r_2_*sampled independently from *U*(0,1); the spiral-search parameters were sampled as *p*∼*U*(0,1)and *l*∼*U*(−,11). All candidate solutions were restricted to the coded design domain −,1+1]^3^. These settings were kept constant across the 20 independent runs of each method.

In terms of hypervolume, GP-MOBO achieved the highest average performance with an HV_mean of 0.9700, followed by PSO (0.9392), WOA (0.9323), Random sampling (0.9203), and GA (0.9120). Because the objective values were normalized to a common [0,1] space and evaluated using the same reference point, these HV values are directly comparable across methods. GP-MOBO also exhibited the lowest variability in hypervolume, with an HV_std of 0.0025, compared with 0.0169 for PSO, 0.0211 for WOA, 0.0224 for Random sampling, and 0.0196 for GA. This indicates that GP-MOBO not only achieved the highest overall Pareto-front quality but also produced the most consistent performance across repeated runs. The ParetoPoints_mean values also differed among methods. GP-MOBO produced the largest average number of non-dominated solutions (40.4), followed by WOA (22.0), GA (18.85), PSO (13.9), and Random sampling (8.05). However, the number of non-dominated solutions alone should not be interpreted as a direct measure of Pareto-front quality, because this metric is sensitive to the density and distribution of candidate solutions generated by each search strategy. For this reason, hypervolume was retained as the primary performance criterion, as it jointly reflects both convergence toward high-performing regions and the extent of the dominated objective space covered by the non-dominated set. A similar pattern was observed for the maximum objective values obtained on the surrogate surface. GP-MOBO achieved the highest average Max_TPC value of 16.5489, followed by PSO (16.4836), WOA (16.4678), Random sampling (16.4566), and GA (16.4385). Likewise, the highest average Max_DPPH value was obtained by GP-MOBO (4.0615), followed by PSO (4.0407), WOA (4.0346), Random sampling (4.0252), and GA (4.0127). The standard deviations associated with these peak values were also smallest for GP-MOBO, further supporting the stability of the GP-guided search strategy.

Overall, these results indicate that GP-MOBO provided the strongest and most stable surrogate-level multi-objective search performance among the evaluated methods. Nevertheless, the HV-NFE convergence analysis should be interpreted as a comparison of computational search efficiency on the fitted surrogate models rather than direct experimental sample efficiency, because the function evaluations correspond to surrogate evaluations rather than additional physical extraction experiments.

The convergence trends presented in Figure 1 further demonstrate differences in the computational search behavior of the evaluated methods on the fitted surrogate models.

**FIGURE 1:**
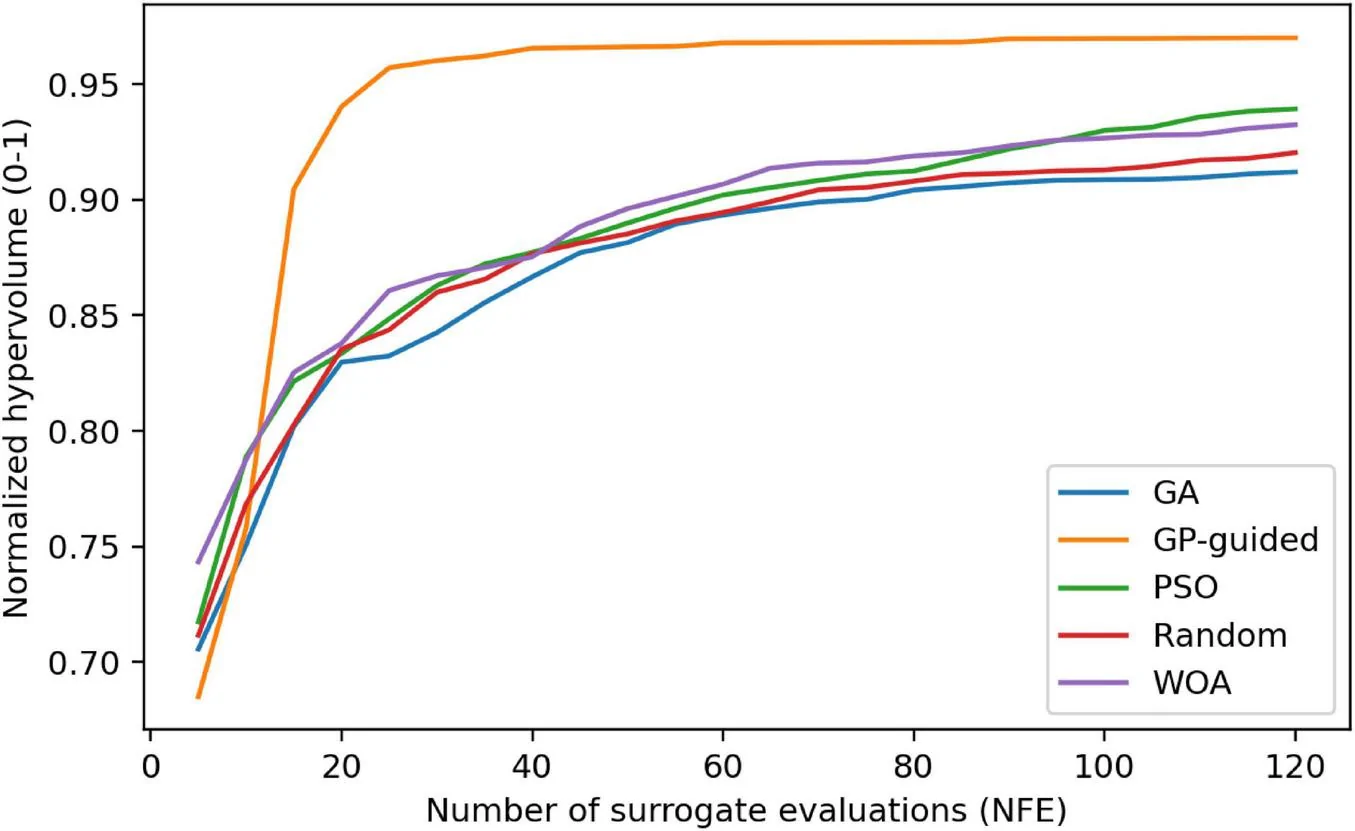
Convergence of normalized hypervolume as a function of the number of surrogate-function evaluations (NFE) for GP-MOBO, GA, PSO, WOA, and Random sampling. All methods were evaluated on the same fitted GP surrogate objective surfaces using common normalization bounds and a common reference point. NFE denotes computational surrogate evaluations rather than additional physical experiments.

GP-guided search exhibited a markedly faster increase in normalized hypervolume during the early stages of the search. Its HV exceeded approximately 0.90 within the first 15 surrogate evaluations and increased to approximately 0.96 by 25 evaluations, after which only marginal improvements were observed, with the curve stabilizing near 0.97. In contrast, GA, PSO, WOA, and Random sampling showed more gradual improvements and remained below the final HV attained by the GP-MOBO approach throughout the evaluated budget. Among the alternative methods, PSO and WOA exhibited comparatively stronger late-stage convergence, whereas GA and Random sampling reached lower final HV levels. These results indicate that the GP-guided strategy identified high-quality regions of the surrogate objective space with fewer surrogate-function evaluations and subsequently maintained a consistently higher hypervolume. This behavior is consistent with the use of GP posterior information within the acquisition strategy to balance exploration of uncertain regions and exploitation of promising regions. Importantly, the HV–NFE curves represent computational search efficiency on the fitted GP surrogate models rather than experimental sample efficiency, because NFE denotes surrogate-function evaluations and not additional physical extraction experiments. Accordingly, Figure 1 should not be interpreted as evidence that the same Pareto quality was experimentally achieved with fewer laboratory trials. Instead, it demonstrates that the GP-guided search explored the fitted surrogate objective space more efficiently than GA, PSO, WOA, and Random sampling under the same computational evaluation budget. Based on this surrogate-guided search, promising operating conditions were subsequently selected for experimental consideration, with both posterior means and predictive standard deviations reported to explicitly account for model uncertainty in the candidate-selection process.

### 3.2 Gaussian process multi-objective Bayesian optimization (GP-MOBO) results

Figure 2 shows the distribution of the current experimental points on the TPC and DPPH axes and marks the non-dominated points. The primary purpose of this visualization is to clearly demonstrate the multi-objective nature of the problem: considering two outputs together rather than a single output can reveal situations where TPC increases while DPPH is constrained, or vice versa. The Pareto chart is a standard reporting element in multi-objective optimization because it objectively defines the “best equilibrium points” from the data.

**FIGURE 2:**
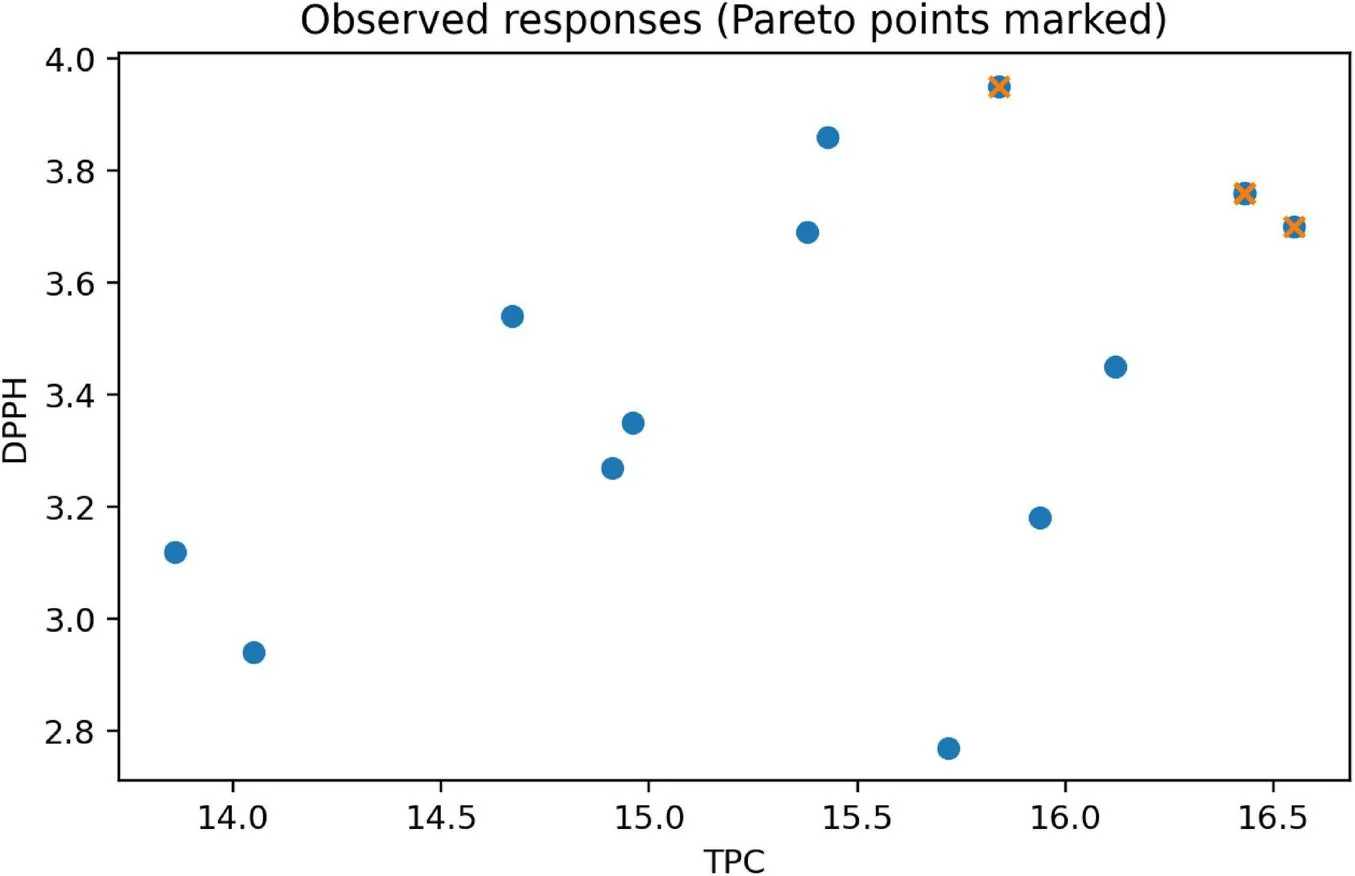
Observed Pareto distribution.

Figure 3 shows the posterior mean (expected value) estimates of the function learned by Gaussian Process regression in the three-factor experimental space for both outputs (TPC and DPPH). The GP approach creates a flexible and non-linear response surface based on the observed points, without being constrained to a parametric polynomial structure. Therefore, the contour maps reflect not only the individual effects of the factors but also the interaction behavior between two factors in a data-driven manner. In the panels, the axes represent coded factor levels (−1: low, 0: center, + 1: high); the color scale indicates the GP mean estimate of the corresponding response.

**FIGURE 3:**
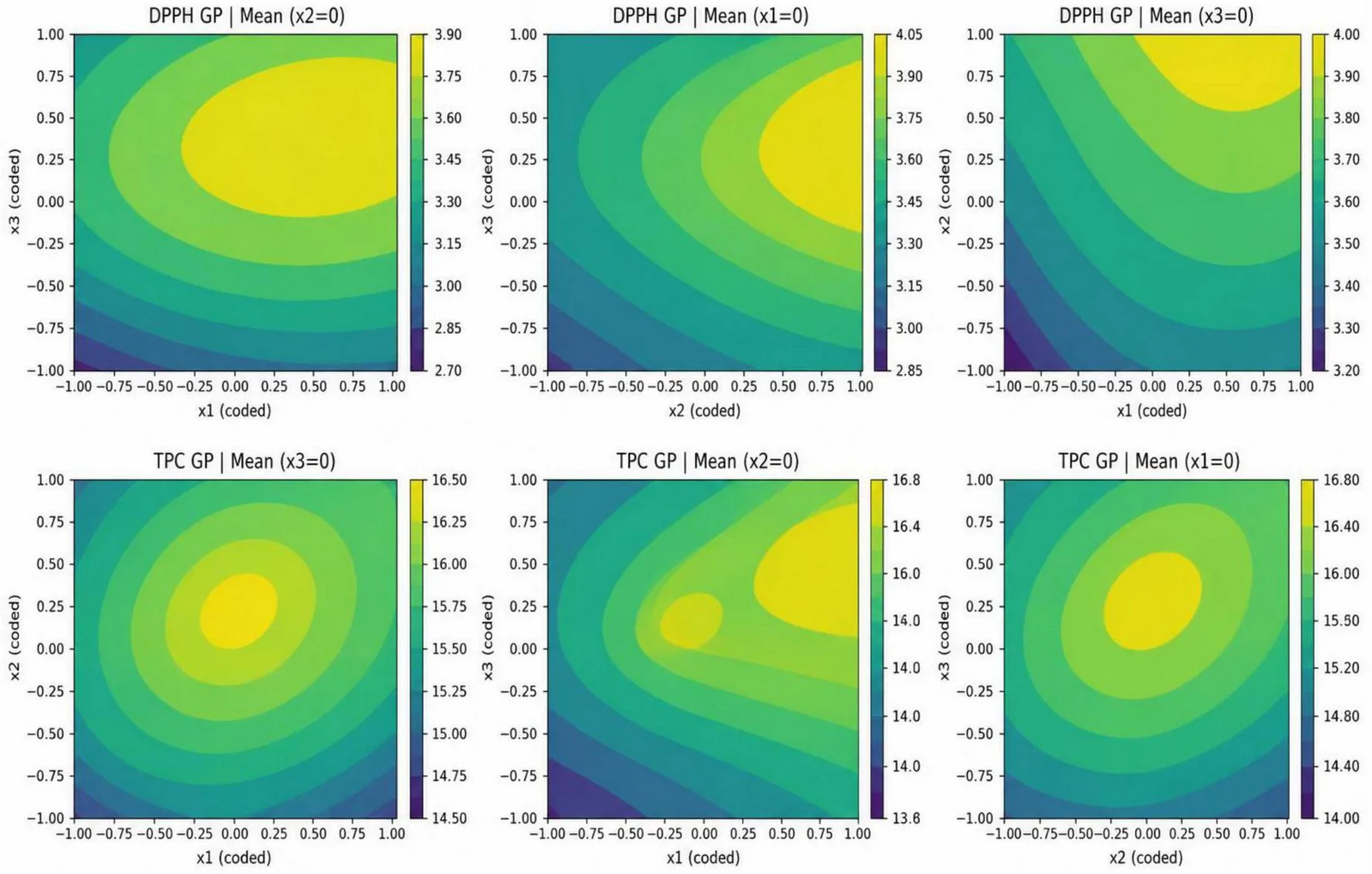
Two-dimensional cross-sections of response surfaces (posterior mean) predicted using Gaussian Process (GP) models: center cross-sections in the coded factor space (x1, x2, x3) for TPC and DPPH.

In the TPC response surfaces, the highest predicted values are generally concentrated around the central-to-moderately positive coded regions rather than at the extreme corners of the design space. In the x1–x2 plane with x3 fixed at zero, a broad elliptical high-response region is observed around the center, indicating that relatively balanced combinations of amplitude and extraction time are favorable for TPC. In the x1–x3 and x2–x3 sections, the location of the high-response region shifts toward moderately positive factor levels, suggesting that TPC is influenced by the joint adjustment of the extraction variables rather than by a single dominant factor. The curved and asymmetric contour patterns further indicate non-additive and potentially interactive relationships among the factors.

The DPPH response surfaces show a somewhat different pattern. Across the three cross-sections, predicted DPPH values generally increase toward moderate-to-high positive coded levels, with broader high-response regions extending across the upper portions of the design space. In the x1–x3 plane, the highest predicted DPPH values are concentrated at positive amplitude and moderate-to-high ethanol levels, whereas the x1–x2 and x2–x3 planes show a more gradual directional increase. Compared with TPC, the DPPH surfaces therefore exhibit a stronger gradient-like behavior, although the curvature and changing orientation of the high-response regions across the different sections indicate that the response still depends on combinations of multiple factors.

Taken together, Figure 3 shows that the favorable regions for TPC and DPPH partially overlap but are not identical. TPC tends to favor more balanced, central-to-moderately positive combinations, whereas DPPH generally shifts toward more positive coded regions. This lack of complete overlap supports the multi-objective nature of the optimization problem and explains why a set of Pareto-optimal operating conditions is more appropriate than a single universal optimum. The posterior mean surfaces are therefore useful for identifying promising regions of the design space, but they should be interpreted together with the corresponding GP uncertainty maps. In the GP-MOBO framework, candidate conditions are selected by considering both predicted response levels and posterior uncertainty, thereby preserving the exploration–exploitation balance during multi-objective candidate selection.

Figure 4 visualizes prediction uncertainty, one of the most critical outputs of GP regression. GP models do not produce just a mean (μ) at each point; they also produce the posterior variance/standard deviation (σ^2^/σ), which represents the confidence level of the prediction at that point. This uncertainty increases in areas of the experimental space where data density is low, where the model is “extrapolating,” or where observations are weak in that region.

**FIGURE 4:**
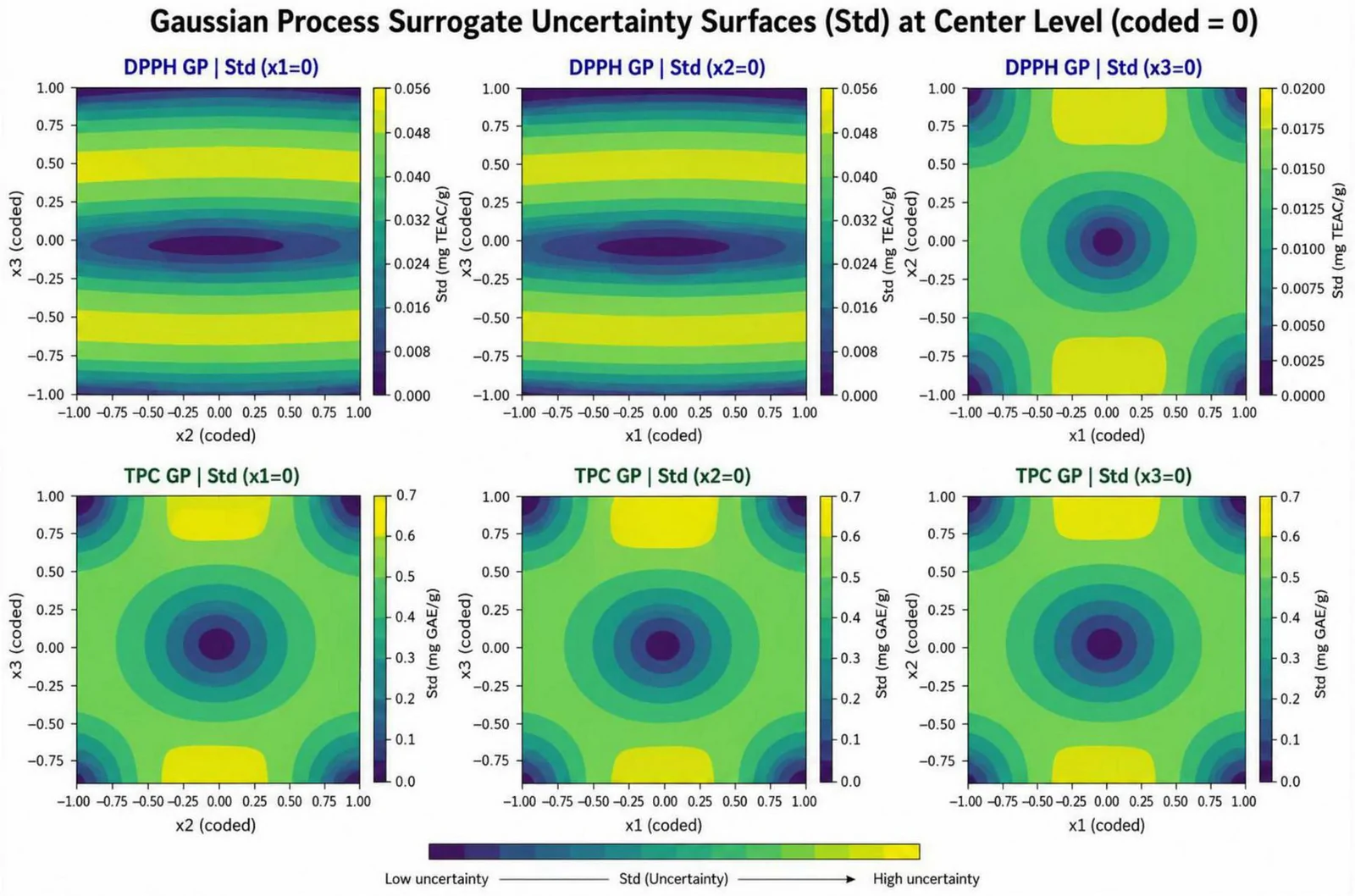
Uncertainty maps (posterior standard deviation, σ) of Gaussian Process (GP) models: Two-dimensional contour plot of prediction uncertainty at the center cross-sections (x1 = 0, x2 = 0, x3 = 0) in the encoded factor space for TPC and DPPH.

For TPC, the posterior uncertainty maps show a clear and consistent spatial pattern across all three center cross-sections. Predictive standard deviation is lowest around the central region of the coded design space and generally increases toward the outer regions. This pattern is consistent with the Box–Behnken design structure, in which the center and edge-midpoint regions are more strongly supported by experimental observations, whereas the full corner combinations of the three-dimensional design space are not directly sampled. Consequently, the GP provides its most confident predictions near the center of the experimental domain, while uncertainty increases as the model moves away from well-supported regions. The approximately concentric and symmetric uncertainty structure observed for TPC indicates that predictive confidence is largely governed by distance from the experimentally supported central region.

From an optimization perspective, these maps help distinguish between regions of high predicted response and regions of high predictive confidence. Low-σ regions, particularly around the central portion of the design space, represent areas where GP predictions can be interpreted with greater confidence. In contrast, candidate conditions located in regions of elevated σ should be treated more cautiously, even when the corresponding posterior mean is high. This distinction is important in the GP-MOBO framework because candidate selection is based not only on predicted response magnitude but also on posterior uncertainty, thereby enabling an explicit exploration–exploitation trade-off.

The DPPH uncertainty surfaces exhibit a more direction-dependent structure than the TPC surfaces. In the sections where x1 or x2 is fixed at zero, uncertainty varies predominantly along the x3 direction, producing broad horizontal bands with alternating low- and high-uncertainty regions. By contrast, in the x1–x2 plane with x3 fixed at zero, the uncertainty structure is more centrally organized, with relatively low uncertainty near the center and higher uncertainty toward portions of the surrounding design space. These differences indicate that predictive uncertainty for DPPH is not distributed uniformly across factor directions.

The anisotropic appearance of the DPPH uncertainty maps may reflect differences in how strongly the available experimental observations constrain the surrogate model along different combinations of factors. In particular, the banded patterns suggest that some directions in the coded design space are supported more consistently by the Box–Behnken observations than others. However, these maps alone do not establish whether this behavior arises from data coverage, response sensitivity, or the fitted GP correlation structure. Therefore, the observed directional uncertainty should be interpreted as evidence of uneven predictive confidence across the design space rather than as direct proof of a specific underlying mechanism.

Overall, the uncertainty maps complement the posterior mean surfaces by identifying where high-response regions are supported by relatively confident GP predictions and where larger uncertainty remains. This joint interpretation of μ and σ is especially important for GP-MOBO, because promising candidates are not selected solely from regions of high predicted TPC or DPPH, but from regions where expected performance and predictive uncertainty are evaluated together.

This finding indicates that the high μ regions observed in certain sections during DPPH optimization (Figure 3) must be evaluated in conjunction with σ; because the objective is not merely high DPPH, but also high DPPH with a reliable prediction. Figures 3, 4 together demonstrate that posterior mean predictions and predictive uncertainty should be interpreted jointly when prioritizing candidate conditions for multi-objective search. Furthermore, Table 4 provides an experimental reference for identifying promising regions of the design space and for potential future iterative optimization studies. Therefore, the observational Pareto set serves as the fundamental point of comparison in both the discussion section and the validation of the suggested results.

**TABLE 4:** Observed Pareto-optimal experimental runs and corresponding responses.

Amplitude (%)	Time (min)	Ethanol (%)	TPC (mg GAE/g))	DPPH (mg TEAC/g)
50	15	50	16.43	3.76
70	22	50	15.84	3.95
70	15	80	16.55	3.70

TPC, total phenolic content (mg GAE/g); GAE, gallic acid equivalents; DPPH, DPPH free radical scavenging capacity (mg TEAC/g); TEAC, Trolox equivalent antiradical activity.

Table 4 summarizes the experimentally observed Pareto-optimal conditions identified from the initial Box–Behnken design and demonstrates the trade-off between TPC and DPPH. Among the observed conditions, the combination of 70% amplitude, 15 min extraction time, and 80% ethanol produced the highest TPC value (16.55 mg GAE/g), whereas its DPPH response was 3.70 mg TEAC/g. In contrast, the condition comprising 70% amplitude, 22 min extraction time, and 50% ethanol produced the highest DPPH response (3.95 mg TEAC/g), but with a lower TPC value of 15.84 mg GAE/g. The condition with 50% amplitude, 15 min extraction time, and 50% ethanol provided an intermediate compromise, yielding a TPC of 16.43 mg GAE/g and a DPPH response of 3.76 mg TEAC/g.

These results clearly illustrate the multi-objective nature of the extraction problem. Specifically, the experimental condition providing the highest TPC did not simultaneously provide the highest DPPH response, while maximizing DPPH was associated with a reduction in TPC. Therefore, no single experimentally observed condition can be considered universally optimal for both responses. Instead, the non-dominated experimental conditions represent alternative compromises between TPC and DPPH and collectively constitute the observed Pareto set.

The observed Pareto set also provides an experimental reference against which the GP-MOBO candidate solutions can be evaluated. The GP surrogate models were used to explore the continuous design space beyond the discrete combinations examined in the initial Box–Behnken experiments and to identify candidate conditions predicted to provide favorable simultaneous combinations of TPC and DPPH. Accordingly, the objective of GP-MOBO was not simply to reproduce the condition associated with the highest individual TPC or DPPH value, but to identify non-dominated candidate solutions with the potential to extend the observed Pareto region. Any improvement predicted for these new candidates should, however, be regarded as surrogate-based until confirmed experimentally.

Figure 5 illustrates the simultaneous consideration of TPC and DPPH through the Pareto framework rather than through a single optimum point. It demonstrates that GP not only performs surface estimation but also generates new experiment proposals by answering the question “which regions are amenable to improvement?” in a multi-objective space. Thus, the study goes beyond classical experimental design reporting to offer a data-driven optimization framework. Table 5 reports the new experimental points proposed by Surrogate-Based Multi-Objective Bayesian Optimization (GP-MOBO), along with the GP’s predicted mean (pred_mean) and predicted standard deviation (pred_std) values for these points. Thus, the recommendations are selected not only based on “high expected performance” but also on an exploration–exploitation balance that incorporates model uncertainty. The EI_scalarized values and objective weights document the scalarization procedure used to explore different trade-off preferences within the multi-objective search. The “Pareto_among_suggestions” field indicates that suggestions are reported by reducing them to a Pareto-optimal subset within themselves, thus eliminating redundant/similar suggestions.

**TABLE 5:** GP-MOBO candidate operating conditions with predicted means, predictive uncertainties, and scalarized acquisition scores.

Variable	S1	S2	S3	S4	S5	S6	S7	S8
Amplitude (%)	58.06	58.54	56.66	56.54	57.53	59.44	55.22	55.71
Time (min)	16.55	18.37	19.85	19.04	19.72	17.08	18.34	17.80
Ethanol (%)	62.04	62.49	58.29	56.82	57.78	61.53	57.63	58.79
x1_coded	0.403	0.427	0.333	0.327	0.377	0.472	0.261	0.285
x2_coded	0.221	0.481	0.693	0.577	0.675	0.298	0.478	0.399
x3_coded	0.401	0.416	0.276	0.227	0.259	0.384	0.254	0.293
TPC pred mean	16.335	16.206	16.092	16.183	16.109	16.296	16.253	16.293
TPC pred std	0.604	0.629	0.627	0.610	0.621	0.617	0.590	0.583
DPPH pred mean	3.897	3.954	3.994	3.964	3.989	3.915	3.943	3.930
DPPH pred std	0.047	0.048	0.037	0.032	0.036	0.046	0.035	0.038
W TPC	0.960	0.769	0.224	0.219	0.262	0.915	0.477	0.708
W DPPH	0.040	0.231	0.776	0.781	0.738	0.085	0.523	0.292
EI scalarized	0.0056	0.0165	0.0342	0.0334	0.0391	0.0088	0.0424	0.0209
Pareto among suggestions	True	True	True	True	True	True	True	True

TPC, total phenolic content (mg GAE/g); GAE, gallic acid equivalent; DPPH, DPPH free radical scavenging capacity (mg TEAC/g); TEAC, Trolox equivalent antiradical activity.

**FIGURE 5:**
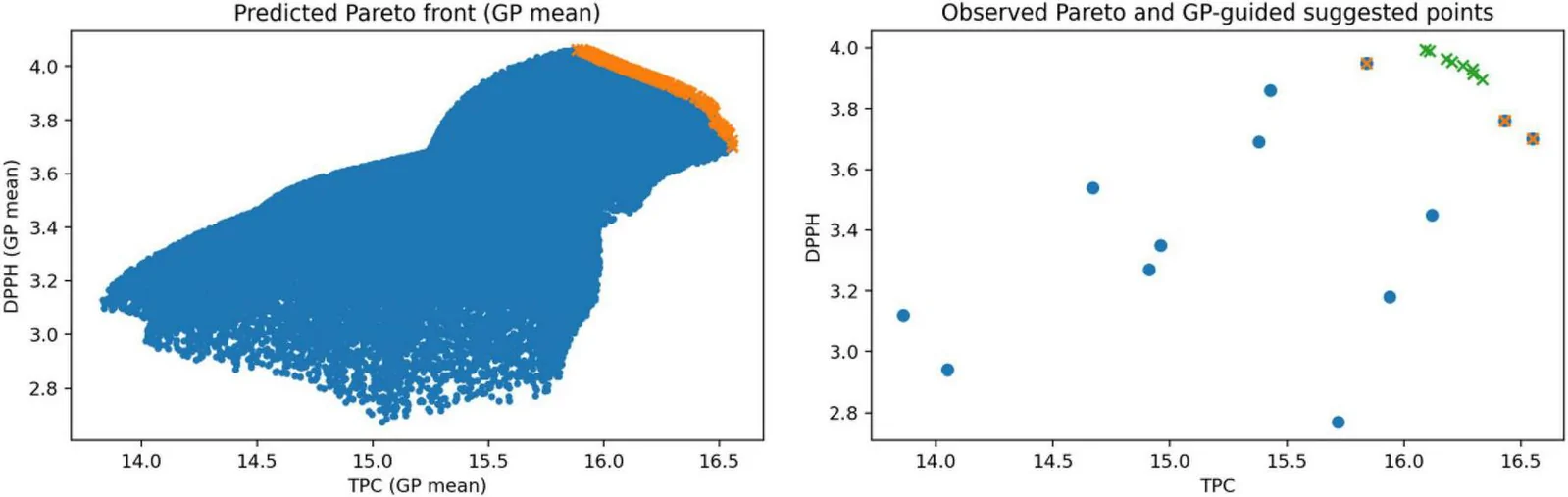
Visualization of GP-based Pareto fronts and candidate optima in TPC–DPPH multi-objective optimisation.

The LOOCV results revealed markedly different predictive performance and uncertainty calibration between the two GP surrogate models (Table 6). The DPPH surrogate showed good out-of-sample predictive performance (*R*^2^ = 0.828), whereas the TPC surrogate yielded a negative cross-validated *R*^2^ (*R*^2^ = −0.640), indicating that its LOOCV point predictions did not outperform a mean-based prediction benchmark. Uncertainty calibration also differed between the responses. For TPC, PICP90 and PICP95 were both 0.867, while the standardized residual dispersion was 1.481, indicating residual under-coverage and an overconfident uncertainty structure. For DPPH, the 95% interval coverage was close to its nominal level (PICP95 = 0.933), although the 90% interval showed under-coverage (PICP90 = 0.800). The standardized residual dispersion of 1.197 suggests better, but not perfect, uncertainty calibration for DPPH. Consequently, DPPH predictions were considered more reliable, whereas TPC predictions and TPC-dependent optimization results were interpreted more cautiously.

**TABLE 6:** LOOCV predictive accuracy and uncertainty calibration of GP surrogates.

Objective	MAE	RMSE	*R* ^2^	PICP90	PICP95	MPIW95	StdResid_std
TPC	0.8291	1.0754	**−0.640**	0.867	0.867	2.462	1.481
DPPH	0.1111	0.1428	**0.828**	0.800	0.933	0.363	1.197

GP, Gaussian Process; TPC, total phenolic content (mg GAE/g); GAE, gallic acid equivalents; DPPH, DPPH free radical scavenging capacity (mg TEAC/g); TEAC, Trolox equivalent antiradical activity; RMSE, root mean square error; MAE, mean absolute error; R^2^, coefficient of determination; LOOCV, leave-one-out cross-validation. Bold values indicate the cross-validated R^2^ coefficients of the GP surrogate models.

The LOOCV analysis revealed substantially different predictive performance between the two GP surrogate models. The DPPH model showed good predictive performance (*R*^2^ = 0.828), whereas the TPC model yielded a negative cross-validated *R*^2^ (*R*^2^ = −0.640). A negative R^2^ indicates that, under leave-one-out validation, the TPC surrogate provided poorer predictions than the corresponding mean-based baseline. Therefore, although the TPC surrogate can be used to characterize the fitted response surface within the investigated design space, its point predictions for unseen conditions should be interpreted cautiously.

The limited out-of-sample predictive accuracy of the TPC surrogate may reduce the robustness of the predicted Pareto front, particularly with respect to the precise ranking and location of candidate solutions along the TPC objective. Future studies may therefore evaluate variance-stabilizing transformations, such as a logarithmic transformation of TPC, where justified by the response distribution, and assess their benefit through cross-validation before surrogate fitting.

### 3.3 Total bioactive compounds

Figure 6A presents the total phenolic content (TPC) of the obtained extracts (mg GAE/g extract), showing that UAEopt resulted in a higher TPC concentration than the NUS. The TPC was determined to be 17.32 mg GAE/g in the UAEopt group and 11.76 mg GAE/g in the NUS group, a statistically significant difference (p < 0.01). The higher TPC yield from UAEopt, despite maintaining the same solvent concentration and contact time, supports the idea that ultrasound application facilitates the transfer of matrix-bound phenolics to the solvent phase, thereby increasing extraction efficiency. While the intermediate values of the conventional extraction groups (CE60/CE120) suggest that extended contact time might contribute to this, the clarity of the fundamental separation in the UAEopt–NUS comparison allows for a more direct demonstration of the contribution of ultrasound application. This enhancement may be attributed to ultrasound-induced structural disruption of the matrix, which facilitates the release of intracellular phenolic compounds and improves their transfer into the extraction medium (19). Similarly, Rueangsri et al. demonstrated that ultrasound-assisted extraction conditions significantly influenced the recovery of phenolic compounds from *Nymphaea pubescens*, with TPC reaching its highest level under the optimized time–temperature combination (34). Altarjami reported that a hybrid ultrasound–microwave-assisted extraction approach enhanced extraction yield and polyphenol recovery from pomegranate peel compared with conventional extraction, further supporting the potential of ultrasound-based processing to improve the recovery of phenolic constituents from complex biological matrices (21).

**FIGURE 6:**
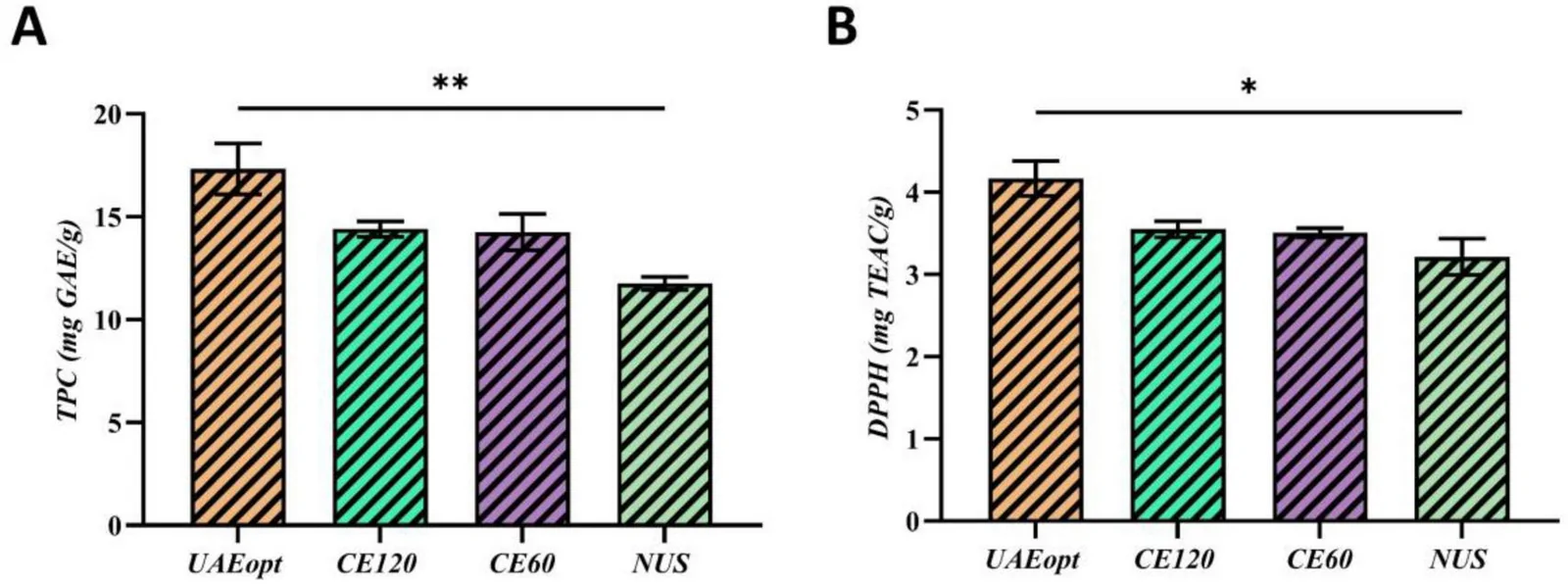
Total phenolic content (TPC) and DPPH free radical scavenging activity of bee bread extracts obtained under different extraction conditions. **(A)** TPC (mg GAE/g) and **(B)** DPPH (mg TEAC/g) for optimized ultrasound-assisted extraction (UAEopt), NUS (same solvent concentration and extraction time as UAEopt), and conventional extraction for 60 min (CE60) or 120 min (CE120). Values are presented as mean ± standard deviation (*n* = 3). Statistical significance for the UAEopt vs. NUS comparison is indicated as *p < 0.05 and **p < 0.01.

New generation extraction methods offer a more environmentally friendly approach by reducing the use of organic solvents, temperature, and time. Bee bread, being a bee product with rich total phenolic content and antiradical activity, is attracting the attention of many researchers and is being investigated from various perspectives. According to these studies, Bayram et al. found total phenolic content in the range of 4.45–7.59 mg GAE/g in their studies using ChCl-Urea extracts supported by ultrasonic extraction conditions (35); Çobanoğlu et al. found it in the range of 5.6–11.4 mg GAE/g in their studies using only ethanol as a solvent (36); Urcan et al. found it in the range of 5.67–12.83 mg GAE/g in their studies using only ethanol as a solvent (37); Can et al. detected a total phenolic content in the range of 4,393–14,917 mg GAE/g (38); In their research in Türkiye, Feizollahi et al. found the values to be in the range of 8.4 ± 0.61 to 26.38 ± 1.83 mg GAE/g. In their research, Gerçek et al. extracted bee bread samples obtained from Turkey using classical methods with 70% ethanol for 50 minutes and determined the total phenolic content to be 4.96 ± 0.54 mg GAE/g (39, 40). In this study, under the optimized ultrasound-assisted extraction condition (UAEopt; 56.5% amplitude, 19.0 min, and 56.8% ethanol (v/v)), the total phenolic content reached 17.32 mg GAE/g, which is higher than values reported in several previous studies. In the present study, in the ultrasound-unassisted experimental model (NUS), levels of 11.76 mg GAE/g were obtained, and these results are consistent with the data of other researchers. Overall, ultrasound-assisted extraction appears to enhance the transfer of total phenolic compounds from bee bread into the solvent phase, resulting in higher total phenolic content in the obtained extracts. In addition, extending the extraction time in conventional extraction also tended to increase the phenolic content of the resulting extracts. The differences in extraction methods, durations, and solvents used in Turkish and other country literature, as well as the lack of studies conducted under identical conditions, highlight the originality of this research.

In Figure 6B, DPPH free radical scavenging capacity is reported in mg TEAC/g, revealing that UAEopt application increased the antioxidant response compared to NUS (UAEopt: 4.17 mg TEAC/g; NUS: 3.215 mg TEAC/g); this increase is statistically significant (*p* < 0.05). The combined evaluation of the TPC and DPPH results indicates that the phenolic enrichment associated with ultrasound application is mirrored by the antiradical activity of the obtained extracts, and that the optimized UAE conditions improve not only the phenolic content but also the functional performance of the extracts. Overall, Figures 6A, B show that the ultrasound-assisted approach can yield a more favorable bioactive profile than NUS under the same extraction time and solvent conditions.

Bee bread, a fermented combination of pollen and honey consumed by honey bees, is notable for its disease-protective properties and health benefits. Numerous studies show that bee bread is a bee product rich in phenolic compounds and therefore has a high antiradical activity. Products with high antiradical activity reduce oxidative stress, thus providing a protective effect against diseases (38). In the present study, the DPPH method was used to determine the antiradical activity of this product. The DPPH free radical scavenging capacity of bee bread was determined by Bayram et al. as 6.41–12.07 mg TEAC/g; by Gerçek et al as 9,83 ± 1,01mg TEAC/g; by Feizollahi et al. 1.41 ± 0.07 to 7.01 ± 0.14 mg TEAC/g; by Çobanoğlu et al. as 1.2–5.5 mg TEAC/g; and by Can et al. as 0.242–2.890 mg/mL (35, 36, 38–40). In the present study, the highest antiradical activity was detected in the ultrasound-assisted extraction method. It appears that ultrasound-assisted applications are preferable for increasing antiradical activity during extraction. Although the results of this study differ from some studies in the literature, they generally align with similar findings. The difference in data is thought to be related to the origin of the bee bread and the variables in the extraction methods.

### 3.4 Phenolic compounds (HPLC)

The phenolic compound profile indicates that the extraction approach significantly influenced both the overall phenolic pool and the compound-specific levels detected in bee bread extracts (Table 6). In terms of total phenolics, UAEopt (44.19 ± 1.23 μg/g) showed the highest level, while CE120 (35.63 ± 0.90 μg/g) and CE60 (31.77 ± 1.15 μg/g) remained at moderate levels; NUS (24.80 ± 0.69 μg/g) yielded the lowest total phenolic content (*p* < 0.05). This pattern suggests that optimized ultrasound-assisted extraction conditions enhanced mass transfer, strengthening the migration of phenolics from the matrix to the solvent phase; however, extraction efficiency was limited under NUS conditions where ultrasound was deactivated. The enhanced recovery of phenolic compounds under UAE conditions may be associated with acoustic cavitation, which can promote structural disruption of the matrix, improve solvent penetration, and facilitate mass transfer between the matrix and the extraction medium (18).

At the individual compound level, gallic acid appears to be the “driving” compound of the phenolic profile (Table 7). Gallic acid reached its highest level in UAEopt at 34.54 ± 0.96 μg/g, while it remained at 22.22 ± 0.62 μg/g in NUS, with a significant difference between the groups (*p* < 0.05). Similarly, ferulic acid increased to 3.12 ± 0.09 μg/g in UAEopt; it decreased to 1.12 ± 0.02 μg/g in CE120, while it remained at its lowest levels in CE60 (0.38 ± 0.01 μg/g) and NUS (0.25 ± 0.01 μg/g) (*p* < 0.05). In contrast, method selectivity is noteworthy in some compounds; For example, neohesperidin reached its highest value of 1.68 ± 0.06 μg/g in conventional extraction (CE60), while it remained at 0.92 ± 0.03 μg/g in UAEopt (*p* < 0.05; Table 7). These findings demonstrate that optimized UAE enhances the total phenolic capacity, while conventional conditions can provide a relative advantage in some compounds.

**TABLE 7:** Individual phenolic compound profile and total phenolic content of bee bread extracts obtained under different extraction conditions.

Phenolic compounds (μg/g)	UAEopt	CE120	CE60	NUS
Gallic acid	34.54 ± 0.96^c^	25.48 ± 0.74^ab^	27.37 ± 0.99^b^	22.22 ± 0.62^a^
Protocatechuic acid	0.12 ± 0.00^c^	0.04 ± 0.00^a^	0.12 ± 0.00^c^	0.07 ± 0.00^b^
Catechin	0.69 ± 0.02^b^	0.00 ± 0.00^a^	0.80 ± 0.03^c^	0.63 ± 0.02^b^
Hydroxybenzoic acid	3.03 ± 0.08^b^	6.54 ± 0.19^c^	0.00 ± 0.00^a^	0.00 ± 0.00^a^
Vanillic acid	0.16 ± 0.00^a^	0.13 ± 0.00^a^	0.48 ± 0.02^b^	0.44 ± 0.01^b^
Gentisic acid	0.33 ± 0.01^b^	0.30 ± 0.01^b^	0.00 ± 0.00^a^	0.00 ± 0.00^a^
*p*-Coumaric acid	n.d	n.d	n.d	n.d
Rutin	0.39 ± 0.01^b^	0.58 ± 0.02^c^	0.00 ± 0.00^a^	0.00 ± 0.00^a^
Ferulic acid	3.12 ± 0.09^c^	1.12 ± 0.02^b^	0.38 ± 0.01^a^	0.25 ± 0.01^a^
Naringin	0.00 ± 0.00^a^	0.13 ± 0.00^b^	0.00 ± 0.00^a^	0.00 ± 0.00^a^
*o*-Coumaric acid	0.17 ± 0.00^c^	0.00 ± 0.00^a^	0.12 ± 0.00^b^	0.00 ± 0.00^a^
Neohesperidin	0.92 ± 0.03^c^	0.37 ± 0.01^a^	1.68 ± 0.06^d^	0.75 ± 0.02^b^
Coumarin	0.21 ± 0.01^c^	0.20 ± 0.00^c^	0.14 ± 0.01^b^	0.00 ± 0.00^a^
Quercetin	0.19 ± 0.01^b^	0.13 ± 0.00^a^	0.42 ± 0.02^c^	0.18 ± 0.01^b^
*Trans*-Cinnamic acid	n.d	n.d	n.d	n.d
Hesperidin	0.33 ± 0.01^c^	0.60 ± 0.01^d^	0.09 ± 0.00^a^	0.13 ± 0.00^b^
Alizarin	0.00 ± 0.00^a^	0.00 ± 0.00^a^	0.16 ± 0.01^c^	0.13 ± 0.00^b^
Total	44.19 ± 1.23^c^	35.63 ± 0.90^b^	31.77 ± 1.15^b^	24.80 ± 0.69^a^

Values are presented as mean ± standard deviation. Within the same row, means followed by different superscript letters (a–d) are significantly different according to one-way ANOVA followed by Tukey’s multiple comparison test (*p* < 0.05). n.d., not detected; UAEopt, optimized ultrasound-assisted extraction; NUS, (same solvent concentration and extraction time as UAEopt); CE60, conventional extraction (60 min); CE120, conventional extraction (120 min).

Extending the conventional extraction time (CE120) was associated with selective increases in some phenolic subclasses; for example, hydroxybenzoic acid reached its highest level in CE120 at 6.54 ± 0.19 μg/g, while it was found at 3.03 ± 0.08 μg/g in UAEopt and undetectable in CE60 and NUS (*p* < 0.05). Similarly, it was observed at 0.58 ± 0.02 μg/g in routine CE120 and undetectable in CE60 and NUS (*p* < 0.05; Table 7). These results support the idea that time extension can create diffusion/solubility-based advantages in some compounds; however, the optimized UAE approach offers a stronger and more consistent gain in terms of total phenolic capacity. This increase in phenolic enrichment, when evaluated together with the TPC (mg GAE/g) and DPPH (mg TEAC/g) results reported in the “Total bioactives” section, demonstrates that optimized ultrasound conditions not only enhance the chemical composition but also improve antiradical activity.

Phenolic compounds can be found in varying amounts in bee bread. One of the important factors determining their amounts is the method used to obtain the phenolic compounds. In present study, when the total phenolic content was examined, it was observed that the ultrasound combined method gave the best results. The methods and amounts used in the extraction of the phenolic compounds we detected were gallic acid (UAEopt 34.54 ± 0.96- NUS 22.22 ± 0.62 μg/g), protocatechlic acid (UAEopt-CE60 0.12 ± 0.00- CE120 0.04 ± 0.00 μg/g), and rutin (CE120 0.58 ± 0.02- UAEopt 0.39 ± 0.01 μg/g). In their study on determining the individual phenolic profile of bee bread, using Deep Eutectic Solvents (DES choline chloride-acetic acid), ethanol and methanol extraction solutions, they found that Gallic acid ranged from 34.71 to 20.11, 213.48, 440.28 μg/kg, respectively; Protocatechic acid ranged from 1225.38 to 63.85, 1760.35, 2440.18 μg/kg, respectively; *P*-coumaric acid ranged from 6021.96 to 3882.49, 6710.15, 6739.53 μg/kg, respectively; They obtained results ranging from 520.08 to 214.19 μg/kg, specifically 1355.65 and 1314.01 μg/kg. Their studies indicate that phenolic compounds were obtained in higher amounts in ethanol and methanol extracts compared to DES extracts While *p*-coumaric acid (n.d) could not be detected in any of the extraction methods used in this study, Kutlu et al. detected it in varying amounts, and it is thought that the differences in the amounts of other phenolic compounds may be due to differences in the bee bread source and extraction method (41). In the present study, gallic acid was found to be the most important phenolic compound extracted according to the UAEopt, CE120, CE60, and NUS methods. In the study by Bakour et al., it is stated that the phenolic compound obtained using hydromethanol extracts was isorhamnetin-O-hexosyl-O-rutinoside (42). Despite the biochemical transformation that occurs during the fermentation of bee pollen in the hive, it is stated that the phenolic compounds in flowers, bee pollen, and bee bread are unaffected (37, 38). It is also stated that variables such as soil and climate did not affect phenolic compounds in the sample types examined (38). Here, the plant species and the effectiveness of the methods used are key factors.

In the present study, the amount of phenolic compounds obtained from bee bread varied depending on the extraction conditions, including application type, extraction time, and processing parameters. Ultrasound-assisted extraction (UAEopt) may offer certain advantages compared to conventional methods, as it enables extraction at relatively shorter times and lower temperatures. This effect is generally attributed to acoustic cavitation, which may enhance mass transfer and promote the release of phenolic compounds from the matrix. Consequently, the optimized UAE conditions resulted in higher total phenolic content compared to the other tested extraction methods. However, process efficiency depends on several factors, such as ultrasound intensity, duration, and the susceptibility of phenolic compounds to oxidation.

### 3.5 Free amino acids (LC- MS)

Bee bread is a remarkable bee product that meets the protein needs of bees. The protein and amino acid profile of bee bread varies depending on geographical location and plant profile, with protein content ranging from 15.30 to 23.48% according to sources in different countries (43). Bee bread is a functional food that is not only a good source of protein, but also rich in essential and free amino acids and possesses many bio-properties (5). Determining these properties of bee bread is only possible by applying the correct extraction methods. In recent years, innovative and environmentally friendly techniques that are alternatives to conventional extraction methods for foods have been researched and are preferred in food extraction (44). Ultrasound application is also among the new-generation extraction methods and, especially for extracting temperature-sensitive molecules, it allows for minimal loss of biocomponents through low-temperature applications (45).

The amino acid profile in Table 8 shows that the extraction approach significantly affected the total amino acid levels (sum of amino acids) detected in the resulting extracts. The total amino acid content was highest in UAEopt at 23409.32 ± 766.26 μg/g; followed by CE120 (18902.07 ± 857.79 μg/g) and CE60 (17425.33 ± 683.07 μg/g), while NUS (12252.85 ± 360.27 μg/g) remained the lowest (Table 8; *p* < 0.05). This gradient suggests that, under the same solvent and contact time conditions, ultrasound application can enhance mass transfer from the matrix to the solvent phase, improving the extractability of not only the phenolic fraction but also the nitrogenous components. The observed trend of increasing total amino acids with extended extraction time (CE120) in conventional extraction is consistent with diffusion-controlled extraction kinetics; however, the clear superiority of UAEopt reveals the method’s “accelerator” effect. The beneficial effects of ultrasound on proteinaceous components have also been reported in other food matrices. Sara et al. demonstrated that UAE significantly improved protein solubility in pumpkin seed extracts, with the highest solubility obtained after 25 min of sonication. The authors attributed this improvement to increased exposure of hydrophilic groups and reduced molecular aggregation, suggesting that ultrasound can modify the extractability and functional behavior of proteinaceous components (17).

**TABLE 8:** Amino acid profile of bee bread extracts obtained under different extraction conditions (μg/g).

Amino acid	UAEopt	CE120	CE60	NUS
Alanine	1442.66 ± 40^c^	977.46 ± 42.75^b^	1100.40 ± 39.90^b^	695.54 ± 23.99^a^
Arginine	1198 ± 33.22^c^	843.53 ± 36.90^b^	884.35 ± 32.07^b^	532.23 ± 18.36^a^
Aspartic acid	3153.76 ± 87.45^c^	3216.49 ± 140.69^c^	2274.85 ± 82.49^b^	1830.12 ± 63.12^a^
Cystine	106.79 ± 2.96^c^	88.22 ± 3.86^b^	80.80 ± 2.93^b^	67.88 ± 2.34^a^
Glutamic acid	3312.26 ± 91.85^d^	1989.79 ± 87.03^b^	2566.37 ± 93.06^c^	1461.06 ± 30.53^a^
Glycine	934.65 ± 25.92^b^	1010.23 ± 59.53^b^	689.66 ± 25.01^a^	591.21 ± 20.39^a^
Histidine	586.53 ± 16.26^c^	442.26 ± 26.06^b^	428.46 ± 15.54^b^	302.36 ± 10.43^a^
Isoleucine	866.68 ± 24.03^c^	633.63 ± 37.34^b^	621.32 ± 31.87^b^	293.34 ± 10.12^a^
Leucine	1927.65 ± 53.45^c^	1445.83 ± 63.24^b^	1435.46 ± 73.63^b^	840.44 ± 28.99^a^
Lysine	1409.74 ± 58.07^c^	1140.89 ± 49.90^b^	1001.35 ± 51.36^b^	734.87 ± 25.35^a^
Methionine	351.30 ± 14.47^b^	358.86 ± 15.70^b^	347.30 ± 17.81^b^	219.20 ± 7.56^a^
Ornitine	7.28 ± 0.34^d^	6.05 ± 0.37^c^	3.06 ± 0.12^b^	1.51 ± 0.05^a^
Phenylalanine	1126.70 ± 46.41^c^	768.44 ± 33.61^b^	831.46 ± 30.15^b^	498.05 ± 17.18^a^
Proline	2921.84 ± 120.35^b^	3173 ± 138.78^b^	2164.45 ± 78.49^a^	2215.02 ± 31.01^a^
Serine	1195.33 ± 49.24^c^	907.43 ± 39.69^b^	1026.63 ± 37.23^b^	745.08 ± 25.70^a^
Threonine	1017.90 ± 41.93^c^	776.90 ± 33.98^b^	769.55 ± 27.90^b^	532.67 ± 18.37^a^
Tyrosine	668.18 ± 27.52^c^	362.00 ± 15.83^b^	371.31 ± 13.46^b^	216.20 ± 10.34^a^
Valine	1126.81 ± 31.25^c^	712.73 ± 31.18^b^	779.06 ± 28.25^a^	425.20 ± 14.66^b^
Taurine	55.30 ± 1.53^a^	48.38 ± 2.11^a^	49.54 ± 1.80^a^	50.91 ± 1.75^a^
Total	23409.32 ± 766.26^c^	18902.07 ± 857.79^b^	17425.33 ± 683.07^b^	12252.85 ± 360.27^a^

Values are expressed as mean ± standard deviation. Within the same row, means sharing different superscript letters (a–d) differ significantly from each other according to one-way ANOVA followed by Tukey’s multiple comparison test (*p* < 0.05). UAEopt, optimized ultrasound-assisted extraction; NUS (same solvent concentration and extraction time as UAEopt); CE60, conventional extraction (60 min); CE120, conventional extraction (120 min). The total amino acid content was calculated as the sum of the amino acids listed in the table.

At the individual compound level, the most quantitatively dominant components of the amino acid composition were glutamic acid, aspartic acid, and proline (Table 8). Glutamic acid reached its highest level in UAEopt with 3312.26 ± 91.85 μg/g and was significantly different from the other groups (CE60: 2566.37 ± 93.06 μg/g; CE120: 1989.79 ± 87.03 μg/g; NUS: 1461.06 ± 30.53 μg/g; *p* < 0.05). In aspartic acid, CE120 (3216.49 ± 140.69 μg/g) and UAEopt (3153.76 ± 87.45 μg/g) similarly reached high values; CE60 and NUS remained at lower levels (Table 8; *p* < 0.05). The fact that CE120 (3173.00 ± 138.78 μg/g) was close to/sometimes higher than UAEopt (2921.84 ± 120.35 μg/g) in terms of proline suggests that the contribution of time extension may be more pronounced in some amino acids and that the extraction may exhibit compound-specific behavior (Table 8; *p* < 0.05).

There are studies investigating the effects of different extraction methods as alternatives to conventional methods on the amino acid profile. For example, Bayram et al. optimized the choline chloride (ChCl)-urea combination with ultrasound as an alternative to conventional solvents and found the total amino acid profile level to be in the range of 10392.33–20323.48 mg/100 g in the ultrasound-optimized groups, while this level was found to be 6289.16 mg/100 g in the control group where conventional extraction was applied. In this research, similar to the present study, the two amino acids detected at the highest levels in bee bread were aspartic acid and proline (35). In the present study, the total amino acid profile in the UAEopt (optimized ultrasound-assisted extraction) group, which was the main experimental group, was determined to be 23409.32 μg/g, while the total amino acid profile in the NUS group, which did not receive ultrasound, remained at 12252.85 μg/g. As can be understood from this, it is seen that the application of ultrasound, as in other studies, enhances the extraction of nitrogenous compounds. In addition, as in similar studies, the most dominant amino acid profile in the present study was formed by glutamic acid, aspartic acid, and proline. In the study by Žitek Makoter et al. comparing conventional, ultrasonic-assisted and supercritical fluids extraction methods in bee pollen, it was stated that the best conditions for amino acid isolation are at temperatures between 40 and 55 °C and pressures between 300 bar and 350 bar (46). Although these researchers conducted their studies on a different bee product, they support this study’s findings and the superiority of ultrasound-assisted extraction. The use of bee products in the food and pharmaceutical industries is important for increasing the bioavailability of amino acids and other biocomponents through ultrasound-assisted extraction methods. Research on bee bread generally focuses on determining its total phenolic properties and total antioxidant capacity. Studies in Türkiye on determining its amino acid profile are limited, and this research is expected to contribute to the literature due to its originality.

UAEopt appears to offer a generally higher gain profile in terms of essential amino acids. Particularly significant is the UAEopt–NUS split for branched-chain amino acids leucine (UAEopt: 1927.65 ± 53.45 μg/g; NUS: 840.44 ± 28.99 μg/g), isoleucine (UAEopt: 866.68 ± 24.03 μg/g; NUS: 293.34 ± 10.12 μg/g), and valine (UAEopt: 1126.81 ± 31.25 μg/g; NUS: 425.20 ± 14.66 μg/g) (Table 8; *p* < 0.05). A similar superiority is observed in critical essential amino acids such as lysine (UAEopt: 1409.74 ± 58.07 μg/g; NUS: 734.87 ± 25.35 μg/g) and phenylalanine (UAEopt: 1126.70 ± 46.41 μg/g; NUS: 498.05 ± 17.18 μg/g) (Table 8; *p* < 0.05). In contrast, the similarity of taurine values among the groups (approximately in the range of 48–55 μg/g) suggests that the extraction approach may have a more limited effect for this compound. In general, the pattern in Table 8 shows that UAEopt improves the composition of bee bread extract at the “multi-component” level by strengthening both the amino acid fraction and the phenolic fraction; therefore, it shows that functional quality can be supported not only through antioxidants but also through nutritional/nitrogenous components.

### 3.6 Correlation and multivariate relationships

The Pearson correlation heat map in Figure 7A quantitatively demonstrates that the antioxidant response in bee bread extracts is correlated with phenolic enrichment. In particular, a strong and significant positive correlation was found between TPC and DPPH (*r* = 0.983, *p* = 0.0169); this indicates that increasing total phenolic content under different extraction conditions increases antiradical activity in parallel. Among the individual phenolics, gallic acid stood out significantly, showing a high correlation with both TPC (*r* = 0.971, *p* = 0.0288) and DPPH (*r* = 0.982, *p* = 0.0180); ferulic acid also showed a strong correlation with DPPH (*r* = 0.964, *p* = 0.0364). However, some relationships did not reach statistical significance (e.g., TPC–ferulic acid: *r* = 0.910, *p* = 0.0902), suggesting that phenolic contribution may be compound-specific and that the overall antioxidant response is more predominantly shaped by certain “repulsive” phenolics. The heat map also showed a correlation between the amino acid profile and the phenolic/antioxidant axis; TPC showed very high positive correlations with many amino acids (e.g., histidine: *r* = 0.9997, *p* = 0.000322; threonine: *r* = 0.9989, *p* = 0.00113; arginine: *r* = 0.9951, *p* = 0.00491; valine: *r* = 0.9930, *p* = 0.00702; phenylalanine: *r* = 0.9920, *p* = 0.00800). In terms of DPPH, tyrosine showed the strongest correlation (*r* = 0.9981, *p* = 0.00185), followed by histidine (*r* = 0.9818, *p* = 0.0182), cystine (*r* = 0.9805, *p* = 0.0195), and valine (*r* = 0.9756, *p* = 0.0244). This pattern supports the idea that the extraction strategy creates a holistic profile change at the “multi-component” level by simultaneously affecting not only phenolic compounds but also the extractability of amino acids; therefore, the optimization approach (UAEopt) can improve compositional quality together.

**FIGURE 7:**
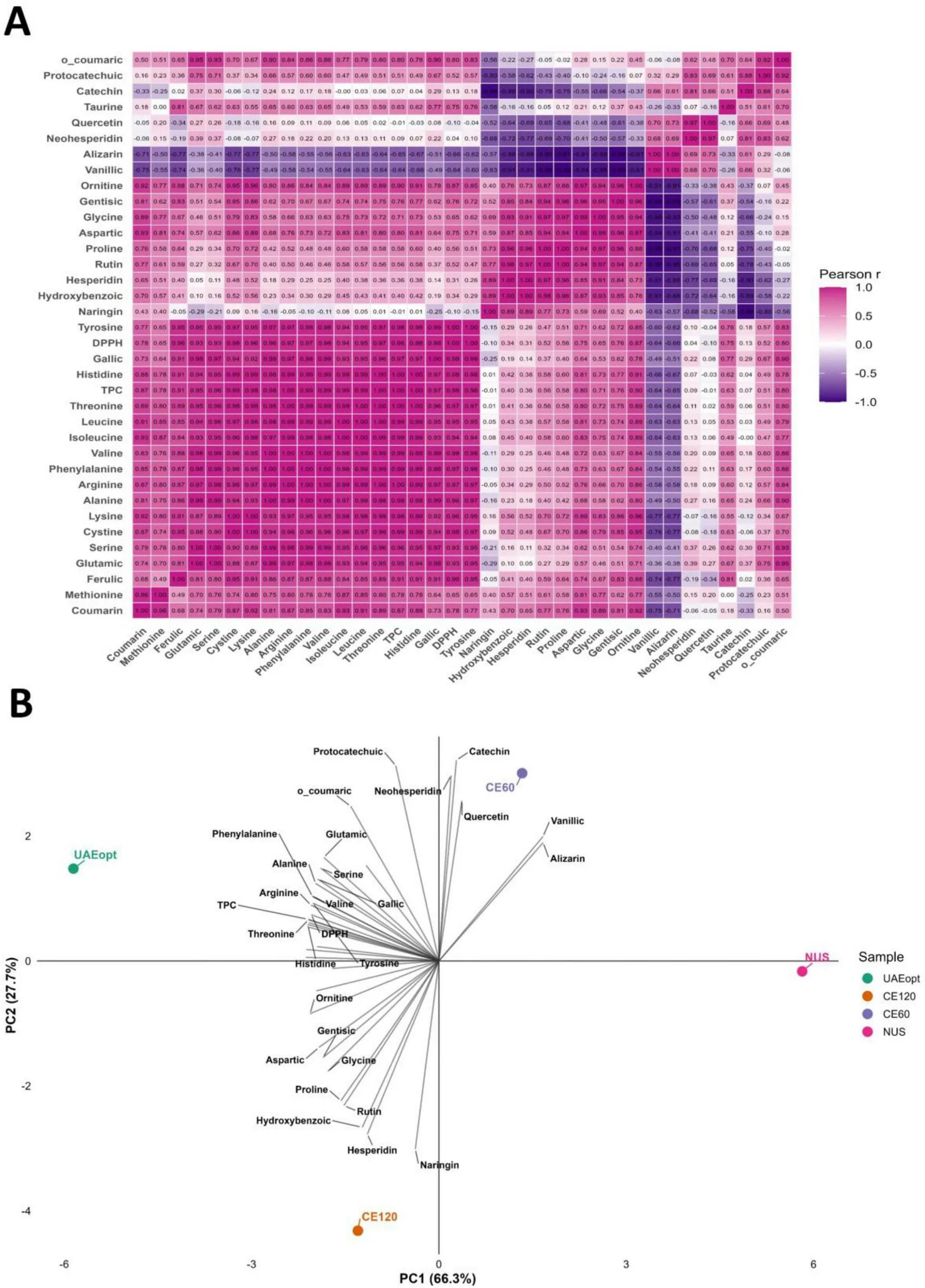
Multivariate relationships among phenolic compounds, amino acids, and antioxidant indices in bee bread extracts obtained under different extraction conditions. **(A)** Pearson correlation heatmap showing pairwise correlation coefficients (Pearson’s *r*) among individual phenolic compounds, amino acids, total phenolic content (TPC), and DPPH free radical scavenging activity. **(B)** PCA biplot illustrating sample separation and variable loadings: PC1 (66.3%) and PC2 (27.7%). Samples are coded as UAEopt (optimized ultrasound-assisted extraction), NUS, CE60 (conventional extraction, 60 min), and CE120 (conventional extraction, 120 min).

The PCA biplot in Figure 7B reinforces the separation between samples by summarizing the multivariate structure under two main components. In PCA, the fact that PC1 explains 66.3% of the variance and PC2 explains 27.7% shows that a very large portion of the total variance can be represented in two dimensions, indicating high interpretability of the model. The clustering of samples (UAEopt, NUS, CE60, CE120) across different regions of the biplot suggests that extraction conditions significantly influence the chemical profile; the orientation revealed by the charge vectors between phenolic compounds/antioxidant indices and amino acids explains the covariation among these parameters. The strong TPC–DPPH and selected phenolic–amino acid relationships observed in Pearson analysis, supporting the separation in the PCA space as well, increase the consistency of the results; thus, reinforcing the conclusion that optimized ultrasound conditions (UAEopt) can create a compositionally more advantageous extract profile compared to conventional and non-ultrasound approaches.

## 4 Conclusion

In conclusion, this study demonstrates the applicability of a single-cycle Gaussian Process-based multi-objective candidate-selection framework for identifying promising ultrasound-assisted extraction conditions for bee bread. By considering predicted responses together with predictive uncertainty, the framework identified a balanced candidate condition that was subsequently validated experimentally. Experimental validation showed that the selected UAE condition increased both responses compared with the matched NUS. HPLC–DAD analysis further demonstrated greater recovery of quantified phenolic compounds, particularly gallic and ferulic acids, while LC–MS analysis revealed the highest total free amino acid concentration among the evaluated extraction conditions, with glutamic acid, aspartic acid, and proline as the predominant components. These findings indicate that optimized ultrasound-assisted extraction can enhance the recovery of multiple bioactive fractions from bee bread within a relatively short processing time. However, the conclusions are limited to the studied bee bread composite and laboratory-scale conditions. Further research should validate the proposed operating conditions using independent bee bread samples from different botanical and geographical origins and evaluate process scalability, energy consumption, compound stability, and the *in vitro* bioaccessibility of the recovered components. Nevertheless, the negative LOOCV R^2^ obtained for the TPC surrogate indicates limited out-of-sample predictive performance for this response. Therefore, TPC-dependent optimization results should be interpreted cautiously, and future studies should evaluate the framework using larger independent datasets and fully sequential surrogate-update/acquisition cycles.

## Data Availability

The raw data supporting the conclusions of this article will be made available by the authors, without undue reservation.
